# A Web- and Mobile App–Based Mental Health Promotion Intervention Comparing Email, Short Message Service, and Videoconferencing Support for a Healthy Cohort: Randomized Comparative Study

**DOI:** 10.2196/15592

**Published:** 2020-01-06

**Authors:** Melanie Elise Renfrew, Darren Peter Morton, Jason Kyle Morton, Jason Scott Hinze, Peter James Beamish, Geraldine Przybylko, Bevan Adrian Craig

**Affiliations:** 1 Lifestyle and Health Research Centre Avondale University College Cooranbong, New South Wales Australia

**Keywords:** guidance, health promotion, eHealth, short message service, videoconferencing

## Abstract

**Background:**

The rapid increase in mental health disorders has prompted a call for greater focus on mental health promotion and primary prevention. Web- and mobile app–based interventions present a scalable opportunity. Little is known about the influence of human support on the outcomes of these interventions.

**Objective:**

This study aimed to compare the influence of 3 modes of human support on the outcomes (ie, mental health, vitality, depression, anxiety, stress, life satisfaction, and flourishing) of a 10-week, Web- and mobile app–based, lifestyle-focused mental health promotion intervention among a healthy adult cohort.

**Methods:**

Participants were recruited voluntarily using a combination of online and offline advertising. They were randomized, unblinded into 3 groups differentiated by human support mode: Group 1 (n=201): standard—fully automated emails (S); Group 2 (n=202): standard plus personalized SMS (S+pSMS); and Group 3 (n=202): standard plus weekly videoconferencing support (S+VCS), hosted by 1 trained facilitator. Participants accessed the intervention, including the questionnaire, on a Web-based learning management system or through a mobile app. The questionnaire, administered at pre- and postintervention, contained self-reported measures of mental well-being, including the “mental health” and “vitality” subscales from the Short Form Health Survey-36, Depression Anxiety and Stress Scale-21, Diener Satisfaction With Life Scale (SWLS), and Diener Flourishing Scale.

**Results:**

Of 605 potential participants, 458 (S: n=157, S+pSMS: n=163, and S+VCS: n=138) entered the study by completing registration and the preintervention questionnaire. At post intervention, 320 out of 458 participants (69.9%; S: n=103, S+pSMS: n=114, and S+VCS: n=103) completed the questionnaire. Significant within-group improvements were recorded from pre- to postintervention in all groups and in every outcome measure (*P*≤.001). No significant between-group differences were observed for outcomes in any measure: mental health (*P*=.77), vitality (*P*=.65), depression (*P*=.93), anxiety (*P*=.25), stress (*P*.57), SWLS (*P*=.65), and Flourishing Scale (*P*=.99). Adherence was not significantly different between groups for mean videos watched (*P*=.42) and practical activity engagement (*P*=.71). Participation in videoconference support sessions (VCSSs) was low; 37 out of 103 (35.9%) participants did not attend any VCSSs, and only 19 out of 103 (18.4%) attended 7 or more out of 10 sessions. Stratification within the S+VCS group revealed that those who attended 7 or more VCSSs experienced significantly greater improvements in the domains of mental health (*P*=.006; *d*=0.71), vitality (*P*=.005; *d*=0.73), depression (*P*=.04; *d*=0.54), and life satisfaction (*P*=.046; *d*=0.50) compared with participants who attended less than 7.

**Conclusions:**

A Web- and mobile app–based mental health promotion intervention enhanced domains of mental well-being among a healthy cohort, irrespective of human support. Low attendance at VCSSs hindered the ability to make meaningful between-group comparisons. Supplementing the intervention with VCSSs might improve outcomes when attendance is optimized.

**Trial Registration:**

Australian New Zealand Clinical Trials Registry (ANZCTR): 12619001009101; http://www.anzctr.org.au/ACTRN12619001009101.aspx

## Introduction

### Background

In 2012, the World Health Assembly requested the development of a plan to address escalating mental distress, resulting in the Mental Health Action Plan 2013-2020, which asserts that there is “no health without mental health” [1]. However, as we approach 2020, mental well-being continues to deteriorate. Depression is the leading cause of disability worldwide [2,3], and its prevalence is rising [4], evidenced by increasing antidepressant use [5] and the high rate of suicide—the second leading cause of death in the 15 to 29 years age group internationally [3]. Comorbidity is common [6], and those with major depression have a 40% to 60% risk of dying prematurely [1]. A 2018 review [7] of 12 countries revealed that depression and anxiety are the third most common reason for visits to a general practitioner in the developed world.

Mental well-being, even in the seemingly healthy population, is compromised by mounting stress and anxiety. In the 2015 Australian Psychological Society’s stress and well-being survey [8], 35% of those surveyed reported significant distress levels, 26% reported above normal anxiety, and 26% described themselves as having moderate to extreme depression levels. Therefore, provision of mental health promotion interventions (MHPIs) may be a vital approach to enhance mental well-being among healthy (ie, nonclinical), community-based cohorts.

There is a growing evidence base supporting the efficacy of MHPIs to improve the mental well-being of nonclinical population groups. These include interventions to alleviate stress [9-11], mindfulness training [12-15], lifestyle medicine strategies such as nutrition [16,17], and exercise [18,19] and an array of positive psychology interventions [20-22]. A recent analytic review [23] highlighted strategies from the fields of positive psychology and lifestyle medicine that have demonstrated effectiveness in enhancing mental well-being. Combining strategies from both fields, the same author devised a lifestyle-focused, multimodal intervention that has been piloted among university students on 2 occasions [24,25] and more recently as a Web-based randomized controlled trial (RCT) for a healthy community cohort [26]. The Web-based RCT demonstrated significantly greater improvements (*P*<.001) than the control group for all outcomes, with medium effect size improvements for the intervention arm in mental health (*d=*0.52), vitality (*d=*0.56), and stress (*d*=0.45). The same intervention was used for this study.

Web- and mobile app–based technology offers an unprecedented opportunity for disseminating MHPIs to healthy cohorts in community settings. Advantages, when compared with face-to-face interventions, include cost-effectiveness [27,28], accessibility, and scalability [29,30]. Furthermore, up to 80% of potential users are “e-preferers” [31]. However, digital delivery also poses unique challenges (eg, quality control, data control, high dropout attrition, and low adherence) that require creative solutions [32,33]. Notably, provision of human support (ie, guidance) is recognized as a possible modulating influence on adherence and outcomes [33,34].

### Human Support

Human support provision in Web-based interventions has generally being associated with improved adherence and outcomes in clinical cohorts [32,35-39]. A meta-analysis of 12 studies treating depression found effect sizes for studies that included human support were larger (*d*=0.61) in comparison with unsupported studies (*d*=0.25) [38]. Nevertheless, some studies have found that the level of human support does not significantly influence outcomes [40-44]. Furthermore, variability in the provision of human support (eg, mode, intensity, and synchronicity) results in high heterogeneity, which makes comparisons between studies problematic [33,39].

It is imperative to investigate the role of human support among nonclinical groups. Support requirements may be markedly different among healthy cohorts. Symptomatic factors (eg, apathy, weakened motivation, and general malaise) that could impede a clinical cohort from completing a program may be nonexistent or differ considerably for a nonclinical group. Conceivably, MHPIs may prove to be a credible pathway to enhance mental well-being and serve as a vital buffer to protect healthy populations against mental distress [45].

Studies comparing human support factors for healthy cohorts engaging in Web- or mobile app–based mental well-being interventions are scarce, and outcomes are mixed. Several primary prevention studies (all classified as “indicated” prevention) have reported no significant difference between supported and unsupported arms [46-48]. Zarski et al [49] examined 3 support approaches and compared the effects on adherence with a stress management intervention. Monitoring and written feedback improved adherence. However, administrative support (ie, access to a support team for technical assistance) had no positive effect [49]. Allexandre et al [50] compared no support, group support, and group support with added expert clinical support for a stress management intervention. Group support was beneficial, but added clinical care contributed no extra benefit. However, the program content was Web-based, and support was provided face-to-face in a work setting, making comparison problematic. Finally, a review and metanalysis of 23 Web- and computer-based interventions to alleviate stress found that supported interventions demonstrated greater effects on outcomes (*d=*0.64) than unsupported (*d=*0.33) [51].

A confounder when drawing conclusions about the impact of human support on the effectiveness of Web- and computer-based programs is that there are many modes and delivery styles classified as human support, which differ markedly in their resource requirements (eg, time, cost, and intensity). For this study, we chose 3 support modes to compare, based on low (automated emails), medium (personalized SMS messaging), and high (facilitated videoconference) resource requirements.

#### Email Support

Emails are widely used [33] and easily incorporated into a Web-based intervention as an asynchronous, low intensity, low cost support method [52]. Notwithstanding, heterogeneity in the way emails are utilized makes comparison difficult, and results are mixed in clinical settings [46,53-55]. For instance, automated emails, often used as engagement prompts, may be built into the system design and require virtually no monitoring once set up. Conversely, personally tailored, individually written emails require effort and time on behalf of the support person and may be considered a more intense mode of support [52].

#### Short Message Service Support

SMS support varies in intensity depending on the method of dissemination (ie, automated or individualized), however, it is easily accessible and portable [56] through the widespread use of mobile phones. Researchers have used SMS support to aid adherence to medication [57,58], support asthma treatment [59], and promote adherence to healthy lifestyle practices [60] among other uses. A systematic review and meta-analysis of Web-based interventions, that used additional support modes demonstrated that SMS had large effects (*d*=0.81) compared with phone (*d*=0.35) and email (*d*=0.18) [61]. Nevertheless, users may ignore SMS prompts when they are perceived as impersonal, too frequent, or automated [62].

#### Videoconferencing Support

Videoconferencing most closely replicates the face-to-face setting; however, it requires a greater investment of time, cost, and human resources. Notwithstanding, it may provide a feasible, personal, and acceptable mode of support, similar to face-to-face settings, as long as technical assistance is available [63]. Participants value ease of accessibility and are still able to bond as a group despite lack of various communication cues (eg, body language) [63]. Videoconferencing has been successfully used to support caregivers [64], patients with chronic disease [65], new parents [66], and bariatric surgery patients [67], among others.

Identifying the optimum input of human support resources to maximize program effectiveness is an important consideration for researchers designing MHPIs for nonclinical cohorts. This comparative study seeks to add to the evidence base by asking the question: “What is the influence of different modes of human support on the outcomes of a Web- and mobile app–based MHPI for a healthy, community-based cohort?”

## Methods

### Recruitment

Participants were recruited from an Australian and New Zealand faith-based cohort. Advertising, conducted from July to September 2018, included offline marketing through periodicals, magazines, and bulletins of the faith-based organization and online marketing using a combination of website, social media, and email strategies. Advertising material directed potential applicants to a Web page to examine the inclusion criteria (Textbox 1), acquaint themselves with participation expectations, and fill out an enrollment application.

**Figure 1:**
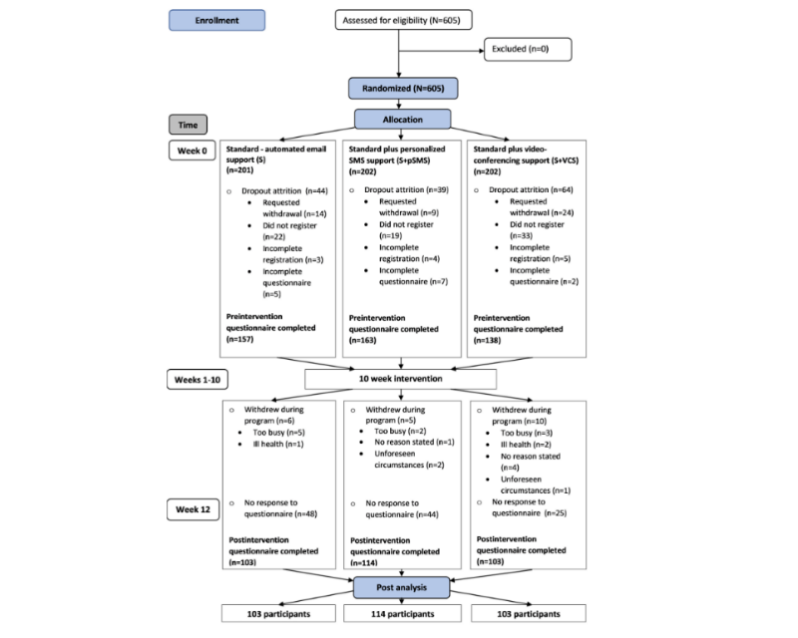
Flow of participants.

Approved applicants were randomized into 1 of 3 intervention groups (Figure 1) using computer-generating software. An email notified applicants of acceptance into the study, group allocation, instructions detailing the steps to complete registration on the Web-based electronic learning management system (eLMS), and a unique link to activate the registration process. Informed consent was gained as part of the registration procedure. Once registered, users could opt to download the mobile app and access all features of the intervention, including self-reported questionnaires, from either the eLMS or from the mobile app.

To provide anonymity, participants were permitted to use a pseudo name for the duration of the intervention, if desired. In the S+VCS group, which involved videoconferencing using the app “Zoom*,*” the participants were given the option to choose a pseudo name and have the camera switched off.

**Inclusion criteria.**

- Aged 18 years or older
- Mobile phone with SMS capability
- Internet access
- Australian or New Zealand resident
- Fluent in English
- Acceptance to provide informed consent
- Permission given for anonymous data to be used for research

### Study Design

The study was a multiarm, randomized comparative design with 3 intervention groups that differed according to modes of human support:

1. Group 1: Standard—fully automated emails (S).
2. Group 2: Standard plus personalized SMS support (S+pSMS).
3. Group 3: Standard plus videoconferencing support (S+VCS).

Figure 1 shows that participants in each group accessed the intervention simultaneously, from September to December 2018.

### Intervention

The 10-week intervention is presented in an audio-visual format and was initially administered in 2017 as a Web-based program on an eLMS. Version 2, released immediately before commencement of this study (August 2018), included an updated eLMS and a mobile app called “MyWellness.” See Multimedia Appendix 1 for screenshots. No new features were introduced during the study period.

The lifestyle-focused intervention is underpinned by the theory of planned behavior (TPB), which has demonstrated substantial health behavior change benefits [61]. The intervention actively seeks to change behavior through education and increase perceived control through easily attainable weekly challenges [24]. Using the pedagogical framework of Learn, Experience, Think, Share, the participants were introduced to basic neuroscience and explored 10 evidence-based strategies from lifestyle medicine and positive psychology for improving mental well-being. Table 1 outlines the weekly topics covered throughout the intervention [68-91].

Participants accessed the intervention free of charge by logging onto the eLMS or the mobile app. Each participant’s personal dashboard provided access to the weekly session, which included approximately 30 min of audio-visual content, a downloadable workbook, and extra reading materials relating to the topic [92]. The intervention emphasized experiential learning by encouraging participants to engage in daily and weekly challenges through which they could log their activity to earn challenge points. Participants were emailed a “timetable overview” (Multimedia Appendix 2), which outlined the recommended schedule to complete the intervention within the 10-week timeframe.

**Table 1:** Intervention overview.

Week	Topic	Overview	Daily challenge	Weekly challenge
1	Speak positively (your limbo is listening)	Limbic system introduction—the “emotional brain” Limbic system is “wired” to language area of brain [68,69]	Offer a genuine compliment.	Memorize an inspirational text or saying.
2	Move dynamically (motion creates emotion)	Proprioceptors (nerve cells that detect movement) pass through the limbic system Movement (even just 10 min) improves mood [70-73]	Complete 30 min of moderate-intensity activity.	Perform resistance exercises once during week (exercises demonstrated on video).
3	Immerse in an uplifting natural environment (blue and green should often be seen)	The limbic system receives messages from all the senses The limbic system likes blue and green spaces (ie, natural settings) The limbic system needs about 30 min of 10,000 LUX of light daily [74-76]	Immerse in an uplifting natural environment for 30 min daily.	Watch a sunrise from an appealing blue or green location.
4	Immerse in a positive social environment (together feels better)	Limbic systems communicate Create positive social environments by making new friends or strengthening existing relationships Forgiveness [77-79]	Daily, do something intentional to show a friend or family member they are loved (use their love language).	Give up your right to hurt someone who has hurt you (Act of forgiveness).
5	Look to the positive (feelings follow your focus)	“Emotional brain” is wired to “Thinking brain” What you focus on affects how you feel Upward or downward spirals [21,45,80-82]	Write down 3 things that went well today “What Went Well?”	Gratitude visit—identify someone you are grateful to, write a gratitude letter to them, deliver and read in person, if possible.
6	Eat nutritiously (food feeds your mood)	Gut bacteria linked to mood Feed gut bacteria high fiber diet Plant-based foods are high fiber Eat a wide variety of fruit, vegetables, legumes, and grains [16,17,83,84]	Eat 8 fists full of fiber daily.	Prepare and share a high-fiber plant-based meal with one or more friends.
7	Rest—sleep (rest to feel your best)	Sleep is fundamental for feeling upbeat (7-8 hours optimal) Blue light vs yellow/orange light Caffeine, lack of physical activity, and blue light—deprived sleep [85,86]	Spend 8 hours in bed every night.	Spend an evening by firelight.
8	Rest—from stress (stress less)	SMILERS strategies “open the valves” Physical activity, practicing mindfulness, laughing, and rest day [13,51,87,88]	15-min “sit in silence” mindful activity.	Take a “guilt-free” day off.
9	Serve others (giving is living)	Contributing/serving is emotionally uplifting Serve sustainably using signature strengths [89-91]	Perform 1 or more random acts of kindness each day.	Use your significant strength to perform a significant act of service.
10	What does it take to flourish?	Five areas to flourish: PEARM—positive emotions, engagement, achievement, relationships, and meaning [90]	Spend time engaging in something you enjoy.	Create a list of goals and an action plan to achieve them.

### Persuasive Systems Design Features

Features of Persuasive Systems Design (PSD) were key to the intervention, and numerous support principles were utilized on the Web platform and mobile app to encourage behavior change. The PSD model, developed by Oinas-Kukkonen and Harjumaa [93], is largely based on earlier work by Fogg [94]. The model outlines a taxonomy of 28 principles, classified under 4 key areas of support, for designing completely digital, behavioral change interventions. “Primary Task Support” components of PSD included the use of “reduction” to simplify key learnings into practical strategies for implementation, along with “self-monitoring” and “rehearsing” desired behaviors in the form of daily and weekly challenges that were recorded on the eLMS or app. An element of “Dialogue Support” [93] utilized was “rewards” in the form of badge icons. “Social Support” elements of PSD [93] provided opportunities for various forms of interaction. Participants could choose to post challenge activity to a public “feed” (viewable by other group members) providing “social learning” and “social facilitation” opportunities. Furthermore, challenges posted as “public” provided opportunity for interaction between group members. “Competition” and “social comparison” were aroused through a challenge points leader board.

### Human Support

Human support was crafted to reflect values of the Supportive Accountability Model (SAM), developed by Mohr et al [34]. They propose a range of support measures to improve adherence, including focusing on process expectations rather than outcome expectations, positive feedback provision, avoidance of controlling behavior, timely communication, being trustworthy, facilitating engaging discussions, and avoidance of pressure tactics [34].

#### Advertising Period

During the advertising period, the mobile app “Hitemup” was used to send enrollees (ie, those who had registered interest in joining the study) weekly, personalized SMS communication to remind them of the upcoming program.

#### Orientation and Registration Period

During orientation week, all enrollees were sent an email link to register onto the eLMS to finalize registration and complete the preintervention questionnaire. A total of 2 SMS reminders were sent, several days apart, as reminders to register.

On the intervention start date, a “welcome” email and SMS were sent to all registered participants outlining the steps to access the first session. In addition, within the first 2 weeks of the intervention, those who had not yet completed registration were sent 4 reminder SMSs and email messages inviting them to access the provided link to complete registration. Once participants completed registration, human support reverted to the constraints outlined for each group, and the eLMS was used to send SMS and automated email messages to registered participants.

#### Group 1 Support: Automated Emails—Standard

Automated emails, which are the routine communication mode outside the research setting, were set up as part of the eLMS. All participants received a weekly email on the day before the next session commencing. The email included a link to a 20 to 25 second video by the presenter, inviting them to engage with the next presentation. A total of 3 days after a lesson was released, the system checked to see if the participant had logged any challenges: if “yes,” participants were sent an email validating their participation, if “no,” an email prompted the participant to complete the relevant challenge. A total of 8 days after a new lesson was released, participants who had not watched the video were sent an email reminder.

#### Group 2 Support: Standard Plus Personalized SMS Messages)

The S+pSMS group received automated emails plus SMS messages that were sent 3 times weekly for the first 3 weeks and then 2 times weekly for the remaining 7 weeks. The reduction from 3 messages per week to 2 messages per week recognized the notion that support may have a threshold [34]; too many messages may be seen as controlling and undermine the commitment of individuals, therefore impairing engagement [34]. The messages focused on process accountability (ie, completing target behaviors such as viewing content and engaging in experiential learning) as opposed to outcome accountability, which may be detrimental and beyond an individual’s control [34].

Each SMS included the participant’s first name, a predetermined message to prompt engagement, and was signed by the research team member (MR) who also provided technical assistance. Specific message content included different combinations of the following: a brief hook sentence (eg, Lesson 2—Learn 3 practical tips to calm your nerves, Lesson 3—Have you had your dose of lux today?), the link to log into the eLMS, an inspiring quote, tips for implementing challenge activity, and a phone number for technical assistance.

#### Group 3 Support: Standard Plus Videoconference Support

As well as automated email support, participants in the S+VCS group were invited to attend a synchronous, videoconference session using the app “Zoom*.*” A weekly timetable (Multimedia Appendix 3) provided 9 different time possibilities, and participants were invited to attend any one of these. During orientation week, participants were encouraged to attend 1 of 9 available “tech check” sessions. Each week, participants were sent 1 reminder SMS and an email with a link to the online meeting. Discussion sessions lasted 20 to 30 min and were led by an experienced educator who holds a post-graduate degree in lifestyle medicine. The facilitator had previously hosted many preventative health videoconferences for participant groups doing the renowned Complete Health Improvement Program. Prior training included 6 mentoring sessions on how to facilitate successful videoconferences in a Web-based setting. The videoconference support sessions included a recap of the weekly content by the facilitator, sharing new learnings and challenge experiences, plus dialogue on ways to incorporate strategies learned into everyday life.

### Measurement—Outcomes

Participants completed a self-report questionnaire, the “7 Dimensions of Wellness Index” (7DWI), at preintervention (week 0) and postintervention (week 12). The 7DWI combines demographic- and lifestyle-related questions with various, freely available, validated instruments that measure 7 well-being domains (emotional, physical, social, spiritual, vocational, intellectual, and environmental). For the purpose of this study, the following instruments were utilized from the 7DWI to measure aspects of mental well-being:

#### 36-Item Short Form Survey

The 36-item Short Form Health Survey (SF-36) is suitable for use in general population surveys [95], and 2 subscales of the SF-36 were used for this study: mental health (5 items) and vitality (4 items) [96]. Good internal consistency and reliability across the whole survey and subscales have been reported [97]. Cronbach alpha scores of .90 for the mental health subscale and .87 for the vitality subscale are well above the minimum reliability standard (.50-.70) [98].

#### 21-Item Depression Anxiety and Stress Scale

The 21-item Depression Anxiety and Stress Scale (DASS-21) is widely used to measure depression, anxiety, and stress (7 items per factor), and satisfactory reliability has been demonstrated for both clinical and nonclinical samples [99-101]. The DASS has demonstrated adequate internal consistency as a total scale (Cronbach alpha >.90) [102] and for the 3 subscales, with Cronbach alpha scores ranging from .76 to .91 [99,101].

#### Satisfaction With Life Scale

Diener 5-item Satisfaction With Life Scale (SWLS) measures global life satisfaction and was initially tested on a nonclinical group of university students [103]. Good internal consistency was found (Cronbach alpha .87) [103], and the SWLS was deemed suitable for a wide range of age groups and settings [104]. Data from 6 studies indicated that the scale has high internal consistency (Cronbach alpha ranged from .79-.89) [105]. Furthermore, a meta-analysis of 62 articles provided 76 reliability coefficients testing the SWLS. The mean Cronbach alpha was .78—signifying good internal consistency [106].

#### Flourishing Scale (Diener)

Diener brief 8-item Flourishing Scale, designed to measure ”social-psychological prosperity,” is suitable for nonclinical cohorts, correlates highly with other measures of well-being, and demonstrates good internal consistency under initial analysis (Cronbach alpha .87) [107]. The Flourishing Scale has a unidimensional factor structure, and more recent analysis, across 2 samples, also showed good consistency (.78 and .83) [108].

### Measurement—Adherence

Primary adherence was measured as the total number of weekly videos viewed out of a possible ten presentations. Challenge adherence was also measured, with participants able to accumulate points through practical daily and weekly challenge activities. Each daily challenge was worth 10 points, and weekly challenges were worth 30 points. Participants could score a maximum of 100 points each week throughout the 10-week intervention, thereby accumulating a total of 1000 points to be considered fully adherent. In addition, for the S+VCS group, videoconference attendance was recorded as a score out of 10.

### Sample Size

The a priori power analysis indicated a required sample size of 148 participants in each arm of the study. This calculation was based on the following assumptions or requirements and relied on data from previous studies [24,26]: (1) participants were to be allocated equally between the 3 arms; (2) an ability to detect a 15% improvement in the mental health and vitality scales, as this was considered a clinically significant outcome [24]; (3) a 30% attrition rate, based on attrition rates observed in a prior similar study [26]; (4) a power level of ≥80%; (5) significance level of 0.05 (95% CI); and (6) a distribution SD of 16.1 in the mental health subscale, based on a prior study [24].

### Randomization

Participants were randomized by a person not on the research team using computer random number generation. Equitable distribution of age and gender was tested and confirmed, negating the need for further stratification. The 3 randomized groups were allocated to a study arm by the person who conducted randomization. Researchers and participants were unblinded, and participants were notified of the mode of support they would receive during registration. As there was no control group, all groups were comparators of interest.

### Statistical Analysis

Data were analyzed using the IBM SPSS Statistics Software (version 25). Within-group changes from baseline to post intervention were calculated using paired *t* tests, and repeated measures generalized linear models were used to measure analysis of variance between groups, taking into account time and group effects. Cohen *d* was used to measure effect size, and Fisher Exact test was used to test for relationships between the categorical variables. The 458 participants who completed the preintervention questionnaire were included in the analysis of baseline characteristics. All remaining analyses included only the participants who completed both preintervention and postintervention measures (n=320). This manuscript was prepared according to Consolidated Standards of Reporting Trials (CONSORT) guidelines [109] and utilized the CONSORT-EHEALTH checklist (Multimedia Appendix 4).

### Ethics and Informed Consent

Ethics approval was granted from the Avondale Human Research Ethics Committee (Approval No. 2018.09). Prospective participants were emailed an “information statement” outlining the details of the study and an “informed consent statement” notifying them that choosing to register onto the eLMS would signify informed consent.

## Results

### Participants

Figure 1 records the flow of participants throughout the intervention period. Potential participants (n=605) were enrolled through the information Web page and were randomized into 3 groups: S (n=202), S+pSMS (n=202), and S+VCS (n=201). A total of 458 participants registered on the eLMS, completed the preintervention questionnaire, and were analyzed for baseline characteristics (S=157, S+pSMS=163, and S+VCS=138). At 12 weeks, 320 out of 458 (69.9%) participants had completed both the pre- and postintervention questionnaire required for postanalysis (S=103, S+pSMS=114, and S+VCS=103).

### Baseline Characteristics

Table 2 describes the demographic and mental health characteristics of the study group at baseline. Fisher Exact tests demonstrated no significant between-group differences in any of the categorical characteristics of the study population. With a mean age of 45.5 (SD 13.7) years, participants were predominantly female (77.7%, 356/458), white (76.9%, 352/458), and had a tertiary education (78.0%, 357/458). There were no significant differences between the groups in any of the psychometric measures at baseline (Table 2).

**Table 2:** Baseline characteristics of the study population.

Factor			Standard automated email group (S; n=157)		Standard plus SMS message group (S+pSMS; n=163)		Standard plus videoconference support group (S+VCS; n=138)		Combined groups (N=458)		Analysis of variance or Fisher Exact test, *P* value
Age (years), mean (SD)			45.6 (13.9)		46.5 (13.4)		44.3 (13.9)		45.5 (13.7)		.19
**Gender, n (%)**											.82
	Female	124 (79.0)		126 (77.3)		106 (76.8)		356 (77.7)			
	Male	33 (21.0)		37 (22.7)		31 (22.5)		101 (22.1)			
	Other	0 (0)		0 (0)		1 (0.7)		1 (0.2)			
**Education, n (%)**											.16
	Primary or elementary	0 (0)		2 (1.2)		0 (0)		2 (0.4)			
	Secondary or high school	36 (22.9)		39 (23.9)		24 (17.4)		99 (21.6)			
	Tertiary undergraduate	74 (47.2)		65 (39.9)		75 (54.3)		214 (46.7)			
	Tertiary postgraduate	47 (29.9)		57 (35.0)		39 (28.3)		143 (31.3)			
**Ethnicity, n (%)**											.34
	White	124 (79.0)		118 (72.4)		110 (79.7)		352 (76.9)			
	Maori/Pacific Islander	12 (7.6)		16 (9.8)		7 (5.1)		35 (7.6)			
	Asian	5 (3.2)		11 (6.7)		2 (1.4)		18 (3.9)			
	Black African/American	4 (2.5)		3 (1.8)		3 (2.2)		10 (2.2)			
	Indigenous	0 (0)		0 (0)		1 (0.7)		1 (0.2)			
	Spanish, Hispanic, Latino	5 (3.2)		3 (1.8)		4 (2.9)		12 (2.6)			
	Other	7 (4.5)		12 (7.5)		11 (8.0)		30 (6.6)			
**Outcome measures, mean (SD)**											
	Mental Health	64.4 (17.3)		65.7 (17.5)		64.6 (16.5)		64.9 (17.1)		>.99	
	Vitality	58.5 (17.4)		60.2 (17.4)		58.6 (17.2)		59.1 (17.3)		.59	
	Depression	3.8 (3.7)		4.0 (4.1)		3.9 (3.5)		3.9 (3.8)		.50	
	Anxiety	2.8 (2.9)		2.5 (2.6)		2.5 (2.8)		2.6 (2.7)		.83	
	Stress	5.7 (3.6)		5.7 (3.6)		5.9 (3.4)		5.7 (3.5)		.67	
	Flourishing	45.2 (7.1)		45.1 (6.8)		44.5 (6.3)		45.0 (6.7)		.10	
	Satisfaction With Life	23.4 (6.9)		22.5 (7.3)		22.5 (7.0)		22.8 (7.1)		.73	

### Measurement—Outcomes

Significant within-group improvements were recorded from pre- to postintervention in all groups and across all domains of mental well-being measured (Table 3). Within-group improvements ranged from medium to large effect sizes (Cohen *d*), with flourishing (*d*=0.64), mental health (*d*=0.67), and vitality (*d*=0.74) demonstrating the largest effect. No significant between-group differences were observed for any of the outcome measures (Table 3).

**Table 3:** Pre- to postchanges in outcome measures of participants and between-group differences.

Outcome Measure			N		Pre, mean (SD)		Post, mean (SD)		Mean change		Change (%)		Within-group change, *P* value		Within-group change, Cohen *d*		Between-group difference*, P* value
**Mental health**																	.77
	Gr 1 standard—Emails	103		65.2 (16.4)		74.8 (15.2)		9.6		14.7		<.001		0.72			
	Gr 2 Standard plus pSMS^a^	114		67.3 (16.7)		76.1 (13.3)		8.8		13.1		<.001		0.69			
	Gr 3 Standard plus VCS^b^	103		66.0 (15.3)		74.2 (14.9)		8.2		12.4		<.001		0.61			
	Combined	320		66.2 (16.1)		75.1 (14.4)		8.9		13.4		<.001		0.67			
**Vitality**																	.65
	Gr 1 Standard—Emails	103		58.7 (16.8)		69.6 (15.8)		10.9		18.6		<.001		0.78			
	Gr 2 Standard plus pSMS	114		61.8 (16.5)		71.9 (13.7)		10.1		16.3		<.001		0.81			
	Gr 3 Standard plus VCS	103		60.1 (16.1)		69.2 (15.4)		9.1		15.1		<.001		0.63			
	Combined	320		60.3 (16.5)		70.3 (15.0)		10.0		16.6		<.001		0.74			
**Depression**																	.93
	Gr 1 Standard—Emails	103		3.5 (3.4)		2.2 (2.6)		−1.3		−37.1		<.001		0.44			
	Gr 2 Standard plus pSMS	114		3.5 (3.5)		2.1 (2.7)		−1.4		−40.0		<.001		0.51			
	Gr 3 Standard plus VCS	103		3.6 (3.3)		2.2 (2.7)		−1.4		−38.9		<.001		0.50			
	Combined	320		3.5 (3.4)		2.2 (2.7)		−1.3		−37.1		<.001		0.48			
**Anxiety**																	.25
	Gr 1 Standard—Emails	103		2.7 (2.6)		1.5 (1.8)		−1.2		−44.4		<.001		0.52			
	Gr 2 Standard plus pSMS	114		2.1 (2.1)		1.4 (1.8)		−0.7		−33.3		<.001		0.35			
	Gr 3 Standard plus VCS	103		2.2 (2.5)		1.3 (1.6)		−0.9		−40.9		<.001		0.43			
	Combined	320		2.3 (2.4)		1.4 (1.7)		−0.9		−39.1		<.001		0.43			
**Stress**																	.57
	Gr 1 Standard—Emails	103		5.8 (3.5)		4.3 (3.1)		−1.5		−25.9		<.001		0.46			
	Gr 2 Standard plus pSMS	114		5.5 (3.4)		4.1 (3.0)		−1.4		−25.5		<.001		0.47			
	Gr 3 Standard plus VCS	103		6.0 (3.2)		4.2 (3.0)		−1.8		−30.0		<.001		0.60			
	Combined	320		5.7 (3.4)		4.2 (3.0)		−1.5		−26.3		<.001		0.51			
**Satisfaction With Life**																	.65
	Gr 1 Standard – Emails	103		23.2 (6.8)		25.6 (6.3)		2.4		10.3		<.001		0.50			
	Gr 2 Standard plus pSMS	114		23.1 (7.2)		26.0 (6.6)		2.9		12.6		<.001		0.64			
	Gr 3 Standard plus VCS	103		22.9 (6.9)		25.5 (6.5)		2.6		11.4		<.001		0.57			
	Combined	320		23.1 (6.9)		25.7 (6.5)		2.6		11.3		<.001		0.58			
**Flourishing**																	.99
	Gr 1 Standard—Emails	103		45.2 (6.7)		48.1 (5.0)		2.9		6.4		<.001		0.61			
	Gr 2 Standard plus pSMS	114		45.1 (7.0)		48.1 (6.1)		3.0		6.7		<.001		0.65			
	Gr 3 Standard plus VCS	103		44.8 (6.2)		47.8 (5.9)		3.0		6.7		<.001		0.64			
	Combined	320		45.0 (6.6)		48.0 (5.7)		3.0		6.7		<.001		0.64			

^a^pSMS: personalized SMS.

^b^VCS: videoconferencing support.

### Measurement—Adherence

Adherence was not significantly different between groups for mean videos watched (*P*=.42) or mean total challenge points scored (*P*=.71). However, there was notable variability in responses as indicated by the large SDs: videos watched out of 10 (S=6.05 (SD 4.0), S+pSMS=6.48 (SD 3.9), S+VCS=6.75 (SD 3.8); challenge scores out of 1000 (S=369 (SD 362), S+pSMS=340 (SD 339), S+VCS=377 (SD 354).

In the S+VCS group, mean VCSS attendance was 2.8 out of 10, and 37 out of 103 participants (35.9%) had zero attendance. Just 19 out of 103 participants (18.4%) attended 7 or more VCSSs. Secondary analysis revealed participants who attended more than 7 VCSSs, compared with those who attended 6 or less, demonstrated significantly greater improvements in the measures of mental health (*P*=.006; *d*=0.71), vitality (*P*=.005; *d*=0.73) depression (*P*=.04; *d*=0.54), and satisfaction with life *(P*=.046; *d*=0.50).

## Discussion

### Principal Findings

This study compared the influence of 3 modes of human support on the outcomes of a Web- and mobile app–based, lifestyle-focused mental health intervention for a healthy adult cohort. Significant improvements in all domains of mental well-being were recorded in all groups, but the mode of human support had no effect. However, attendance at the VCSSs was low, hindering the ability to draw comparisons.

The study population could be classified as healthy (ie, normal), as evidenced by baseline DASS scores that were within the normal range: depression, mean=3.5 (normal 0-4); anxiety, mean=2.1 (normal 0-3); stress, mean=5.7 (normal 0-7). Notably, despite the “healthy” starting point, medium to large effect size improvements were observed. The results of this study are similar to 2 pilot trials [24,25] and an RCT [26] using the same intervention. Medium to large effects may be due, at least in part, to the multimodal nature of the intervention producing a compounding effect [24]. As the intervention embeds a combination of evidence-based strategies from lifestyle medicine and positive psychology for improving mental well-being, the overall effect of the intervention could be expected to exceed that of a single modality approach.

Videoconferencing has been successfully used as a form of support in various settings, but the sessions were not well attended in this study. Previous research highlights the benefits of videoconferencing as a feasible and acceptable mode of support as long as technology assistance is provided [63]. Benefits include enhanced group bonding, peer observation, personal sharing, and a higher social presence when compared with other forms of digital support [63]. However, although videoconferencing has been used as an effective method to support caregivers [64], chronic disease patients [65], new mothers [66], and postsurgical patients [67], it was underutilized in this intervention.

Several factors may have contributed to the underutilization of the VCSSs. First, Web-based interventions are essentially “pull” technologies, relying on the participant to initiate access to the intervention [110]. Conversely, human support features generally “push” participants to engage with a digital intervention [110]. Within this study, automated emails and SMS messages served as “push” strategies, requiring no effort on the part of the participant. However, videoconferencing support is a “pull” device that required participants to actively seek engagement by attending the scheduled session, and this may have been a contributing factor in low engagement. Second, to overcome potential scheduling barriers, 9 sessions were offered within a week, but this did not translate into higher levels of participation and may have negatively impacted development of group dynamics and peer interaction. Participants could choose to attend any of the 9 sessions offered, which meant group bonding, a known videoconference advantage [63], may have been impeded by a lack of continuity in attendance within each time slot. Notwithstanding, a myriad of other factors may have negatively impacted VCSS engagement, such as time constraints, confidence to use technology, privacy or exposure concerns, and perceptions about effectiveness [63,111]. Undoubtedly, adherence is dynamic in nature [56], and many interindividual variations are still unexplained [49]. The low attendance at the VCSSs highlights the need to investigate engagement facilitators and barriers more thoroughly. Given that the majority of the S+VCS group participants (82%) did not engage regularly with the support provided, this group, in effect, received a comparable level of support with the S group (automated emails), hampering between-group comparability.

Stratified within-group analysis in the S+VCS group showed significantly greater improvement in depression, mental health, vitality, and satisfaction with life metrics for those who attended 7 or more VCSSs. Nevertheless, the results need to be treated with caution because the stratified subgroup is self-selected, no longer randomized, and therefore subject to bias. In addition, the small number of participants and unknown contributing factors (eg, motivation) make drawing conclusions problematic. Elucidating the reasons why participants chose to engage, or not, with videoconferencing support would be an important topic for further research.

Despite the lack of influence of human support in this study, the multimodal intervention demonstrated statistically significant improvement across all groups in all outcome measures, with medium to large effect sizes. Strengthened by results of previous pilot studies [24,25] and the RCT in 2017 [26], it may be feasible to trial the lifestyle-based intervention in primary prevention or clinical settings in the future to attenuate symptoms for those who are at high risk or already suffering from a disorder.

### Strengths and Limitations

The intervention, Web design, and additional human support used in this study were underpinned by theoretical models, including the TPB [112], a framework for PSD [93], and key principles of the SAM [34]. Consistency in the VCSSs was enhanced by using 1 experienced, online group facilitator rather than multiple facilitators of varying skill levels. In addition, the study population included a large homogenous cohort, and this was further strengthened by a wide range of age groups from 18 to 81 years.

Limitations included low attendance at VCSSs, which meant that many S+VCS group members probably experienced the intervention similarly to the S group (automated emails), negatively impacting the ability to make meaningful between-group comparisons. Notably, the previous RCT in 2017 [26] demonstrated similar statistically significant improvements within the intervention group to what was seen within the 3 groups of this study. Both the RCT and this study commenced with cohorts who were considered “normal” (indicated by the DASS scores). Conceivably, in adding human support, a ceiling effect was observed, and gauging further improvements above and beyond the benefits of the intervention itself were not realistically measurable.

The cohort were predominantly Seventh-day Adventist church members, which diminishes generalizability to other population groups because of commonly held lifestyle practices (eg, no alcohol or tobacco). Furthermore, participants were skewed toward white females and those who held a tertiary qualification. Although such demographics are commonly portrayed in digital interventions [113,114], these factors limit the generalizability of findings to the broader population. Participants were unblinded, and the study relied on self-reporting for all instruments, which comes with risk of reporting biases (eg, poor recall), and participants are sometimes unaware of their own personal motives and behavior [56].

Other limitations include the failure to measure how participants engaged with automated emails or SMS messages. In addition, we did not gather data regarding the proportion of participants who accessed the program using the mobile app as an alternative to the Web-based platform, which would have been a useful comparison. In future applications, a postintervention survey should include questions regarding the use and preferences for the delivery systems (ie, computer or mobile app), plus participant perceptions regarding the influence of and personal engagement with email and SMS messaging. It would also be important to administer the intervention to a broader population sample to improve generalizability.

### Conclusions

The findings of this study strengthen the rationale for Web- and mobile app–based interventions as easily accessible and scalable mental health promotion initiatives. The study demonstrated that a lifestyle-focused intervention improved the mental well-being of a healthy cohort, irrespective of human support. Low VCSS attendance reduced the ability to draw meaningful between-group comparisons, and hence, the influence of different human support modes, although SMS messages provided no significant benefit over automated email support. Stratified analysis demonstrated that regular attendance at a VCSS might be a possible method to enhance outcomes; however, more research is needed regarding factors that influence engagement with that mode of support.

## Appendix

Multimedia Appendix 1 Website and app screenshots.

Multimedia Appendix 2 Timetable overview.

Multimedia Appendix 3 Videoconference meeting schedule.

Multimedia Appendix 4 CONSORT-EHEALTH checklist (V1.6.1).

## Abbreviations

7DWI: 7 Dimensions of Well-Being Index
CONSORT: Consolidated Standards of Reporting Trials
DASS: Depression, Anxiety and Stress Scale
eLMS: electronic learning management system
MHPI: mental health promotion intervention
PSD: persuasive systems design
RCT: randomized controlled trial
S: standard group
S+pSMS: standard plus personalized short message service group
S+VCS: standard plus videoconferencing support group
SAM: supportive accountability model
SF-36: Short Form Health Survey
SWLS: Satisfaction With Life Scale
TPB: theory of planned behavior
