# Supplementary material for: A Web- and Mobile App–Based Mental Health Promotion Intervention Comparing Email, Short Message Service, and Videoconferencing Support for a Healthy Cohort: Randomized Comparative Study
Source: J Med Internet Res. 2020 Jan 6;22(1):e15592. doi: 10.2196/15592 (PMC6971514; doi:10.2196/15592)
Supplement: Multimedia Appendix 1 [file jmir_v22i1e15592_app1.pptx]

## Slide 1
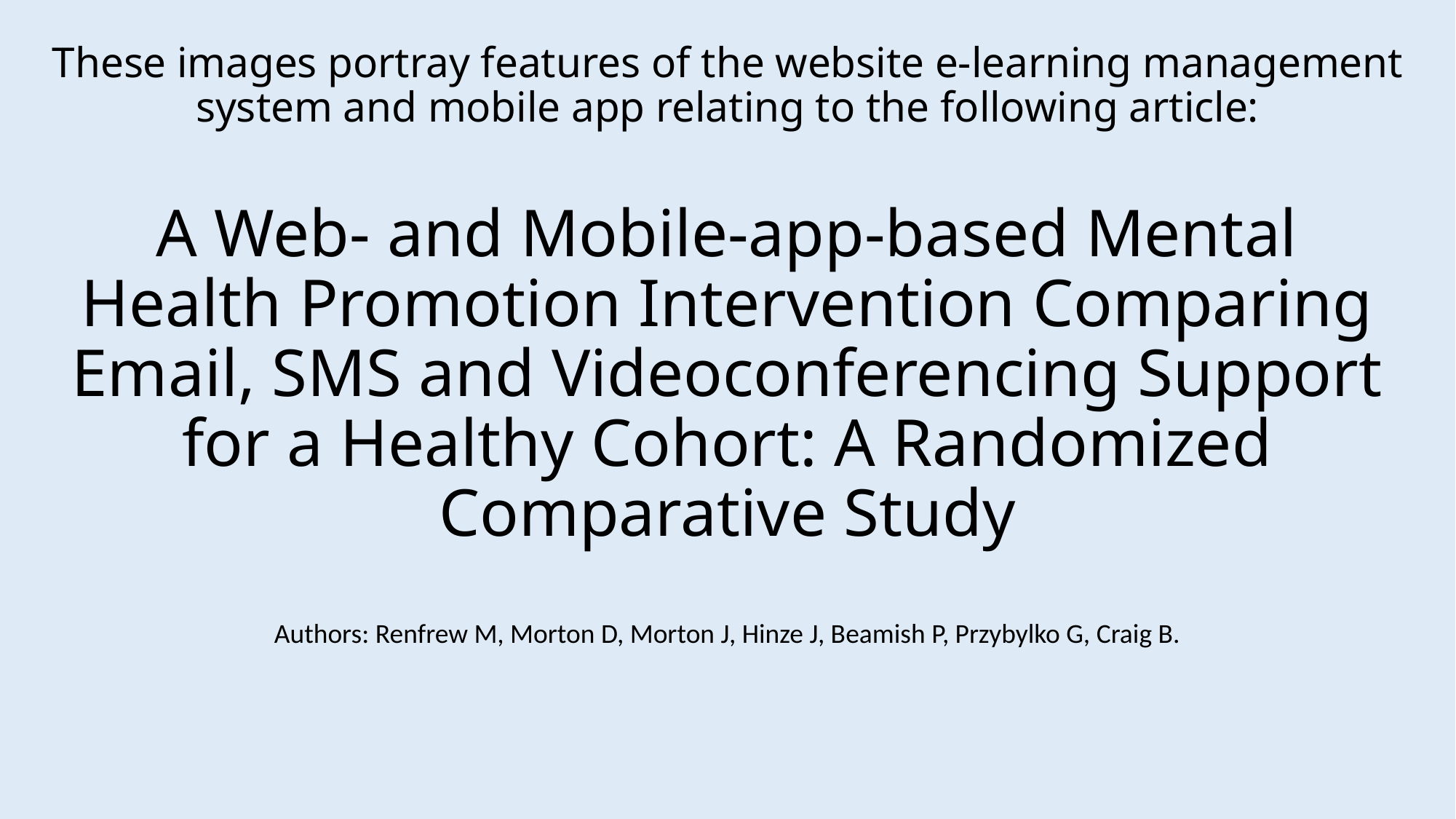

These images portray features of the website e-learning management system and mobile app relating to the following article:
A Web- and Mobile-app-based Mental Health Promotion Intervention Comparing Email, SMS and Videoconferencing Support for a Healthy Cohort: A Randomized Comparative Study
Authors: Renfrew M, Morton D, Morton J, Hinze J, Beamish P, Przybylko G, Craig B.

## Slide 2
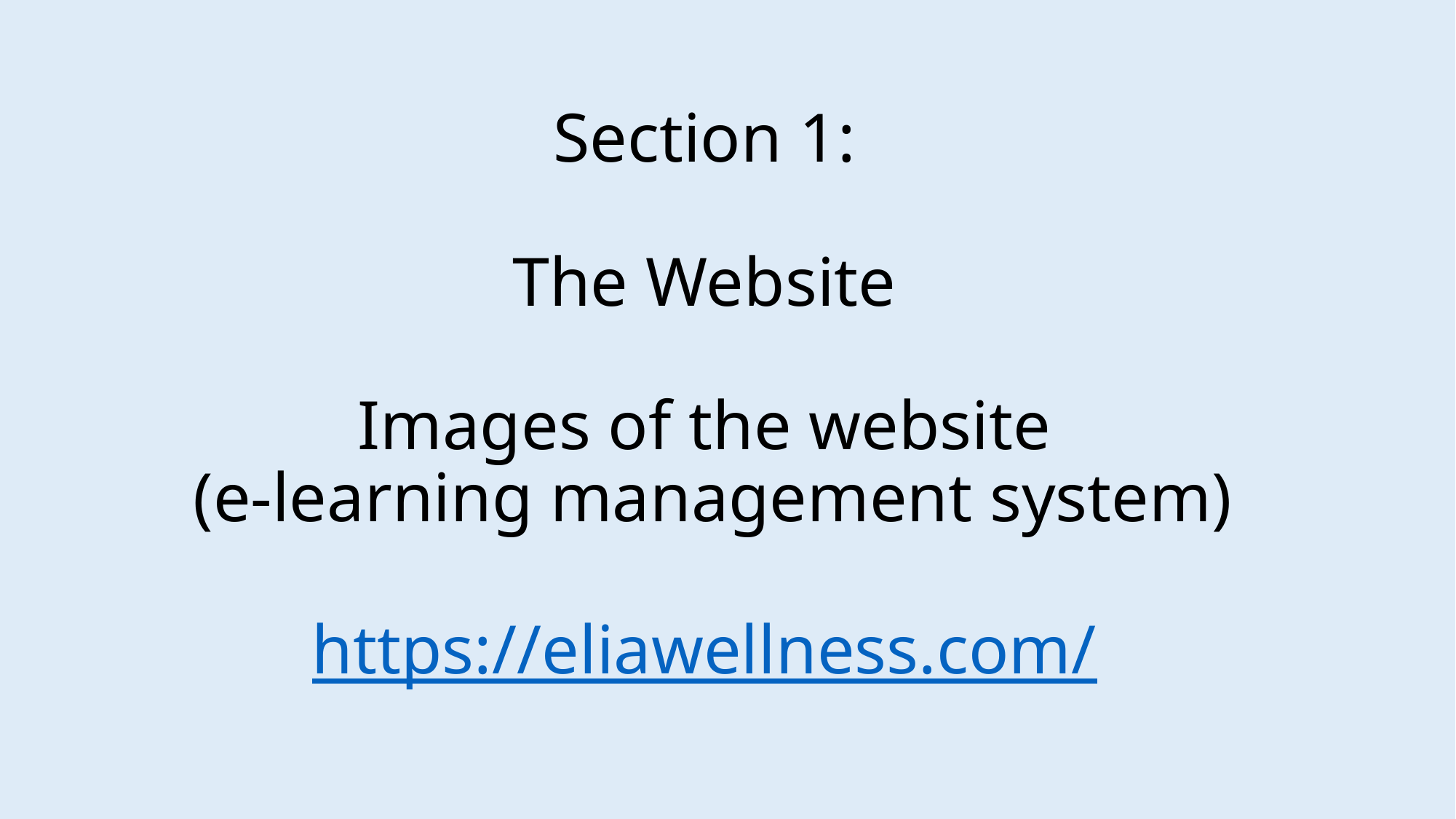

Section 1:
The Website
Images of the website
(e-learning management system)
https://eliawellness.com/

## Slide 3
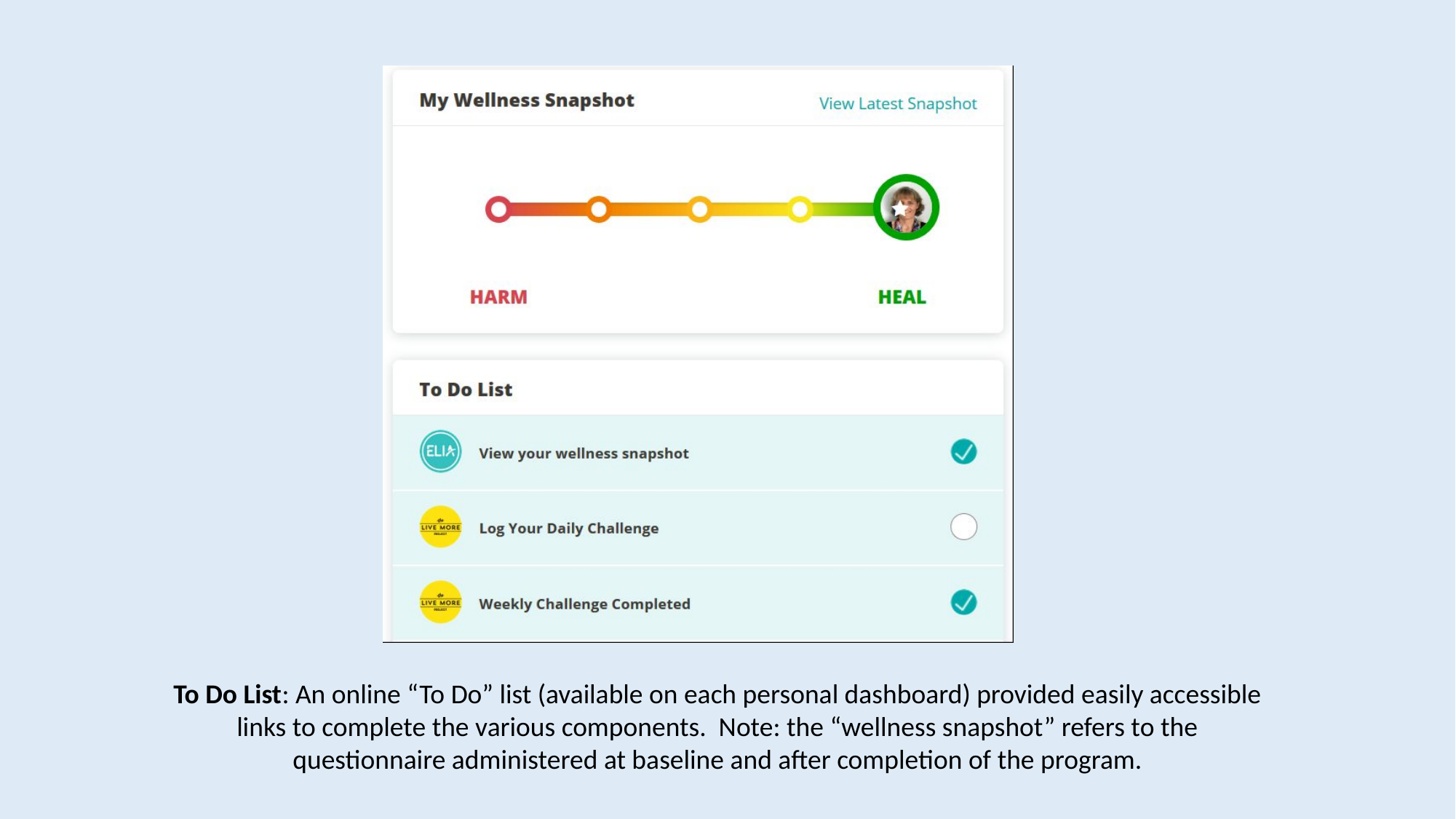

To Do List: An online “To Do” list (available on each personal dashboard) provided easily accessible links to complete the various components. Note: the “wellness snapshot” refers to the questionnaire administered at baseline and after completion of the program.

## Slide 4
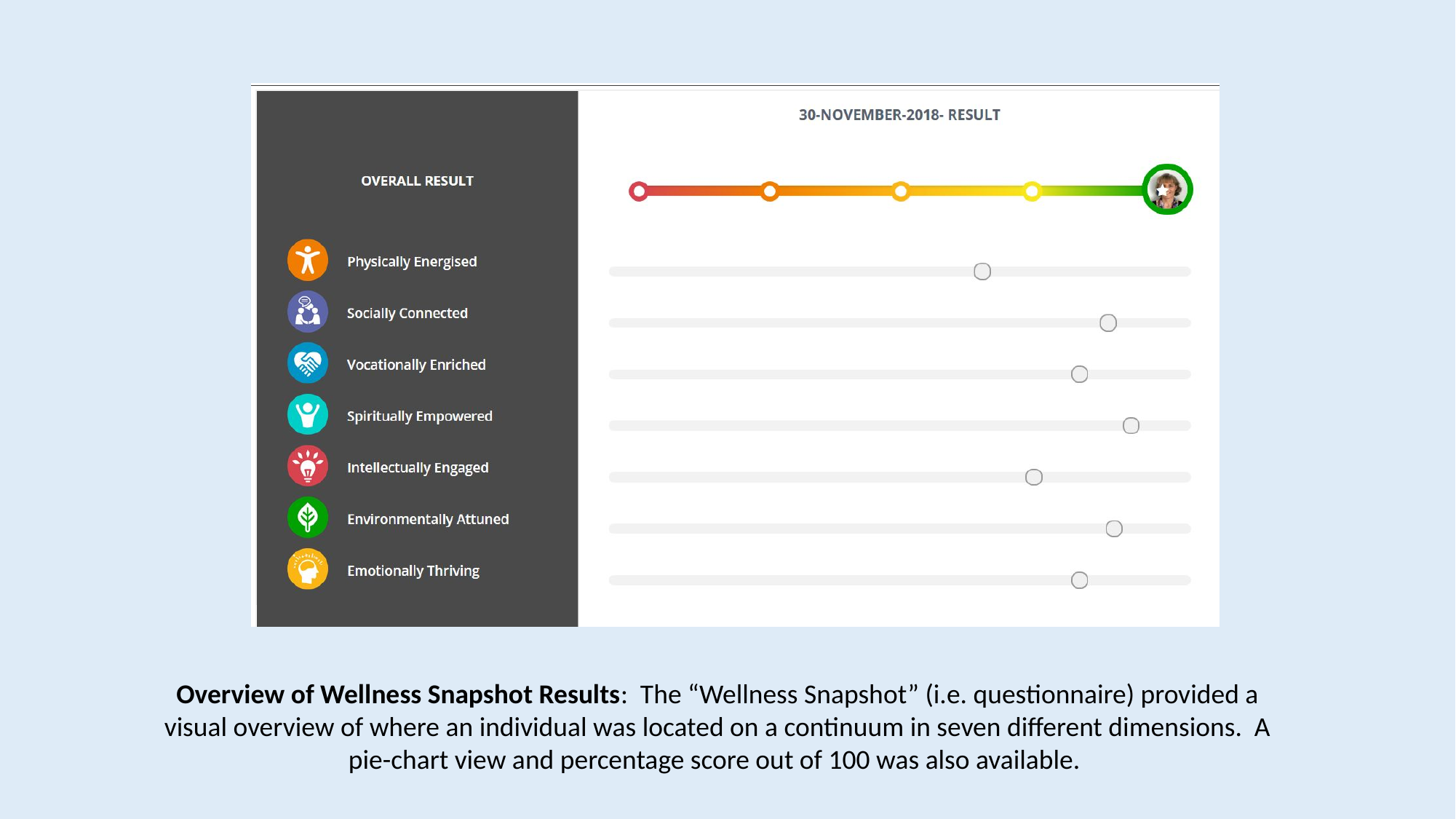

Overview of Wellness Snapshot Results: The “Wellness Snapshot” (i.e. questionnaire) provided a visual overview of where an individual was located on a continuum in seven different dimensions. A pie-chart view and percentage score out of 100 was also available.

## Slide 5
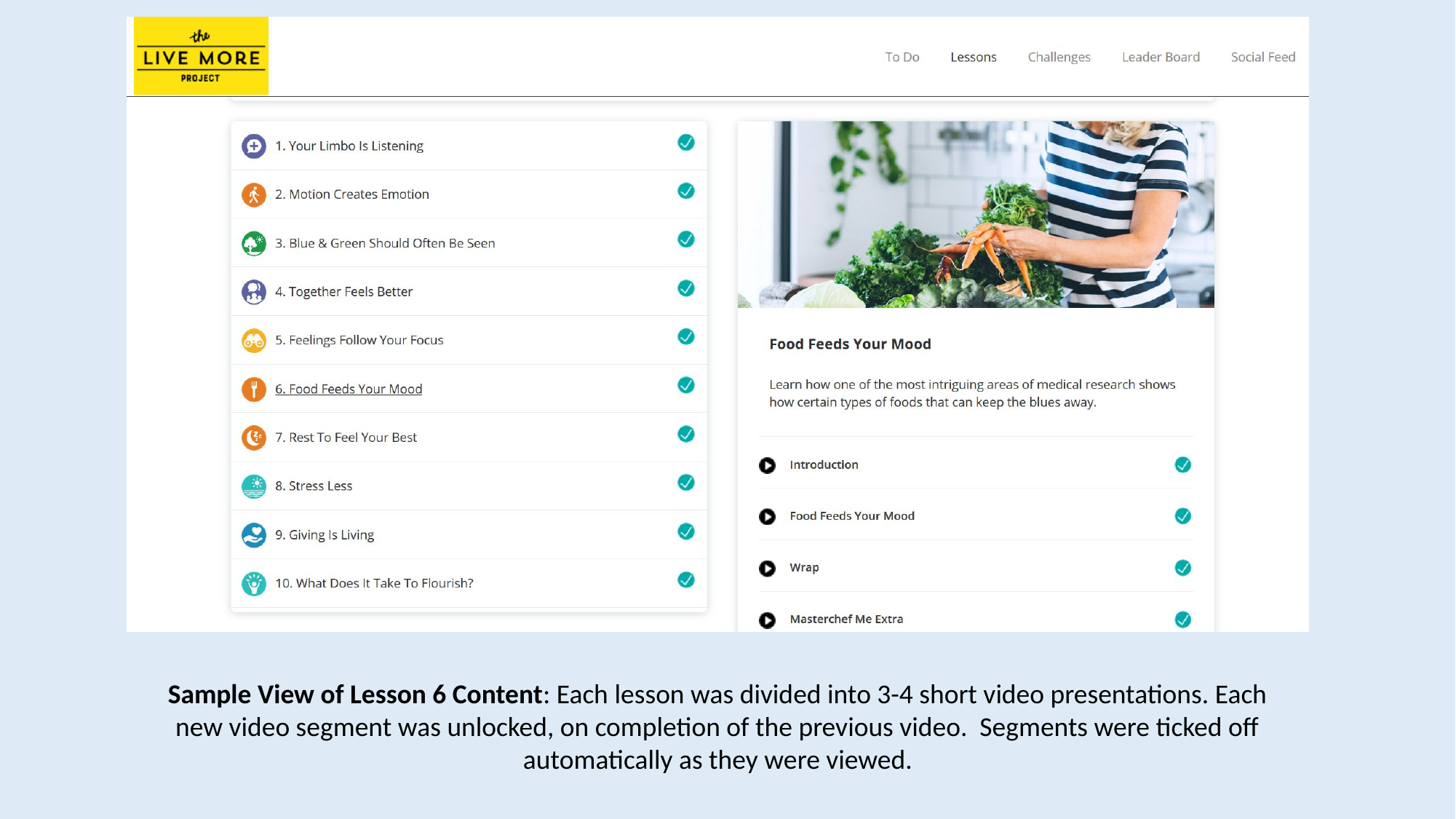

Sample View of Lesson 6 Content: Each lesson was divided into 3-4 short video presentations. Each new video segment was unlocked, on completion of the previous video. Segments were ticked off automatically as they were viewed.

## Slide 6
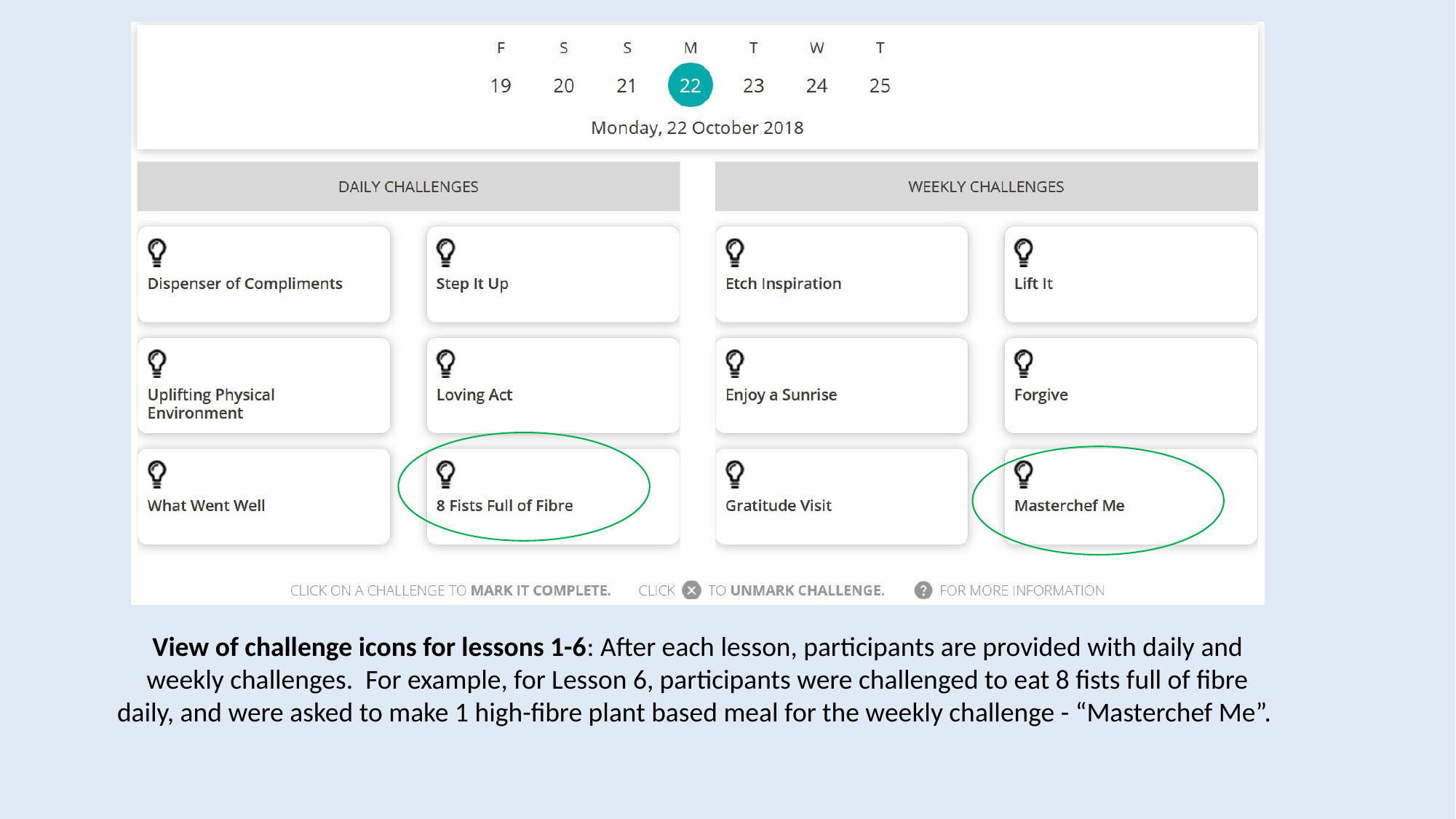

View of challenge icons for lessons 1-6: After each lesson, participants are provided with daily and weekly challenges. For example, for Lesson 6, participants were challenged to eat 8 fists full of fibre daily, and were asked to make 1 high-fibre plant based meal for the weekly challenge - “Masterchef Me”.

## Slide 7
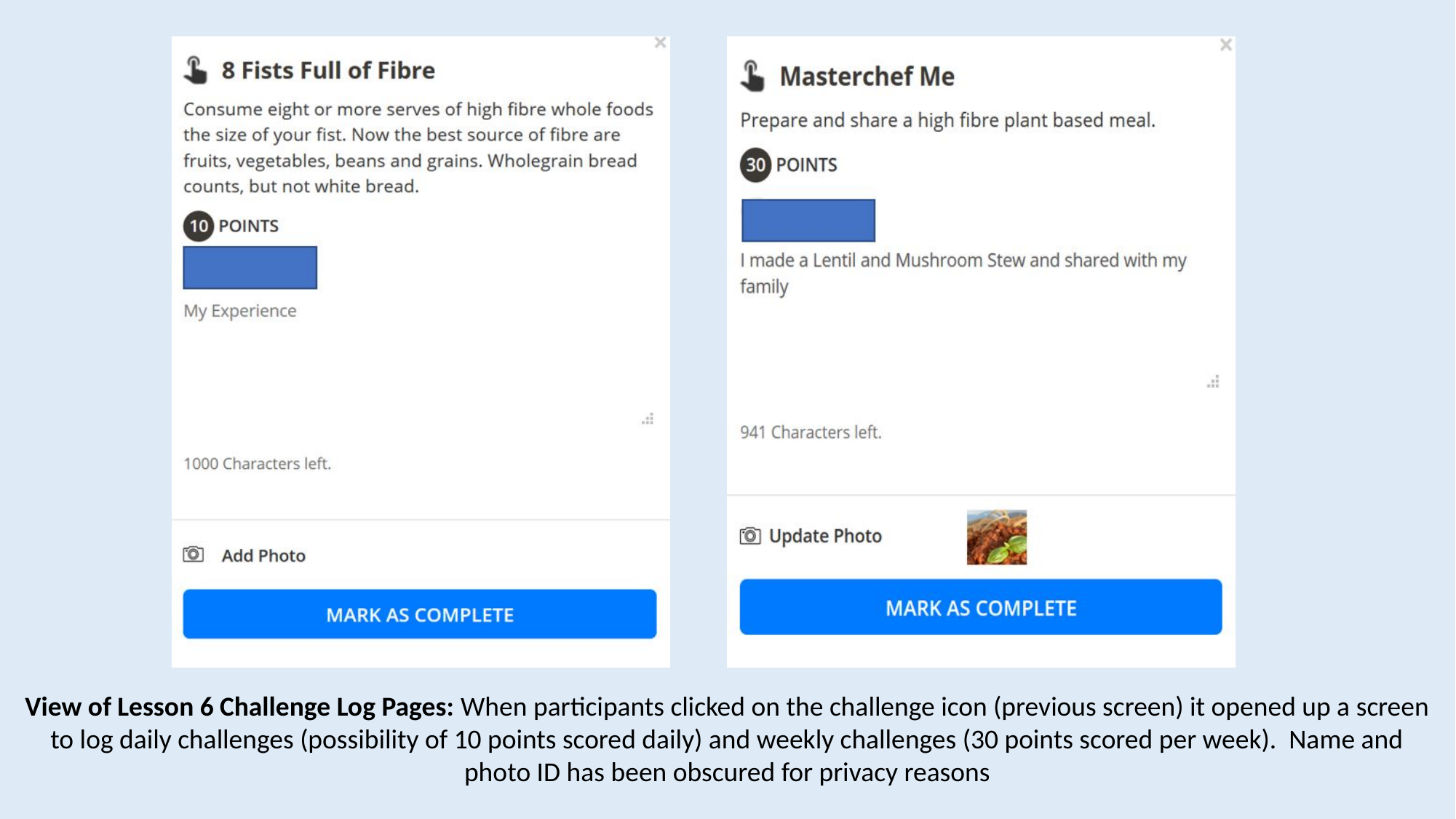

View of Lesson 6 Challenge Log Pages: When participants clicked on the challenge icon (previous screen) it opened up a screen to log daily challenges (possibility of 10 points scored daily) and weekly challenges (30 points scored per week). Name and photo ID has been obscured for privacy reasons

## Slide 8
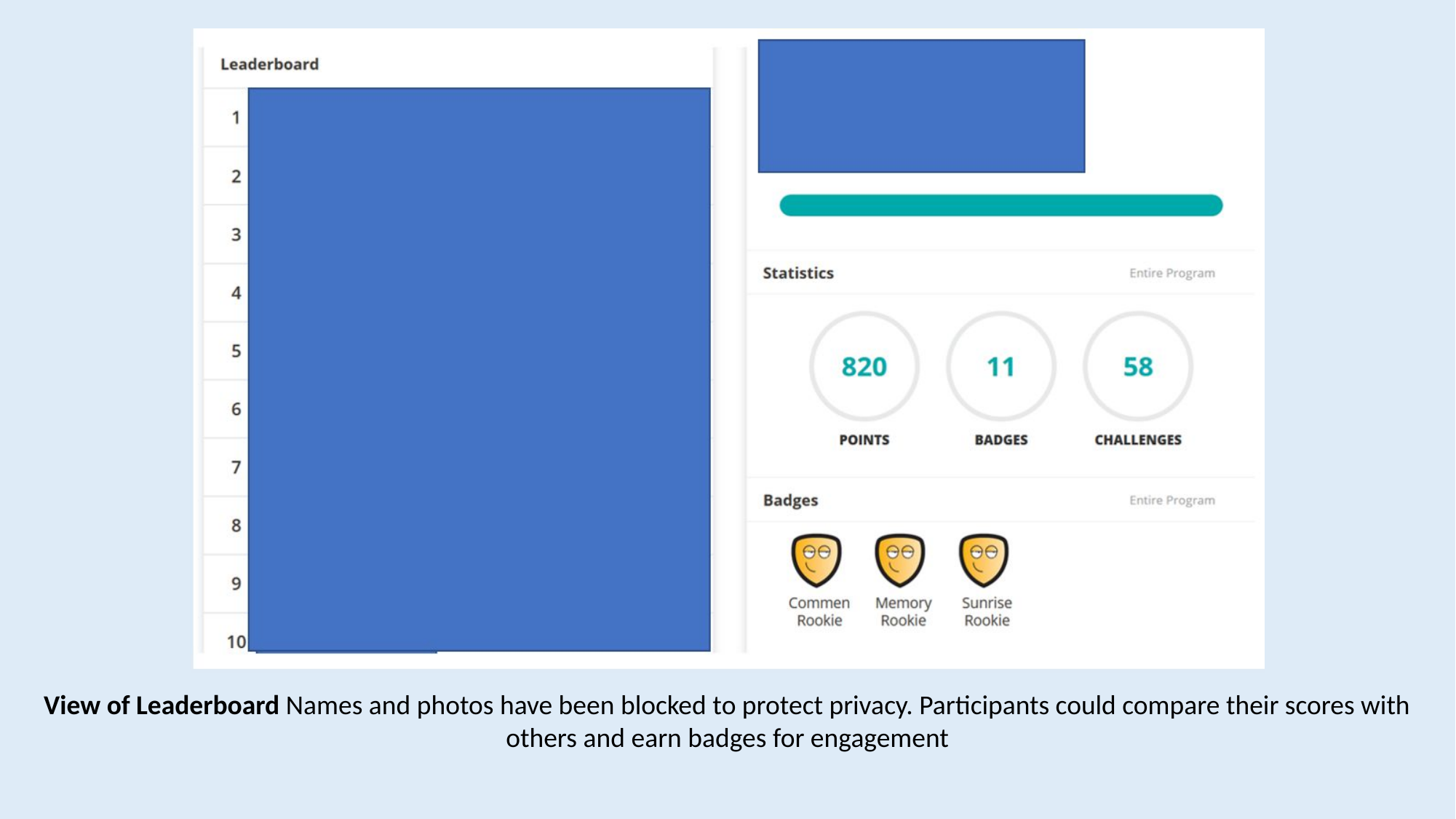

View of Leaderboard Names and photos have been blocked to protect privacy. Participants could compare their scores with others and earn badges for engagement

## Slide 9
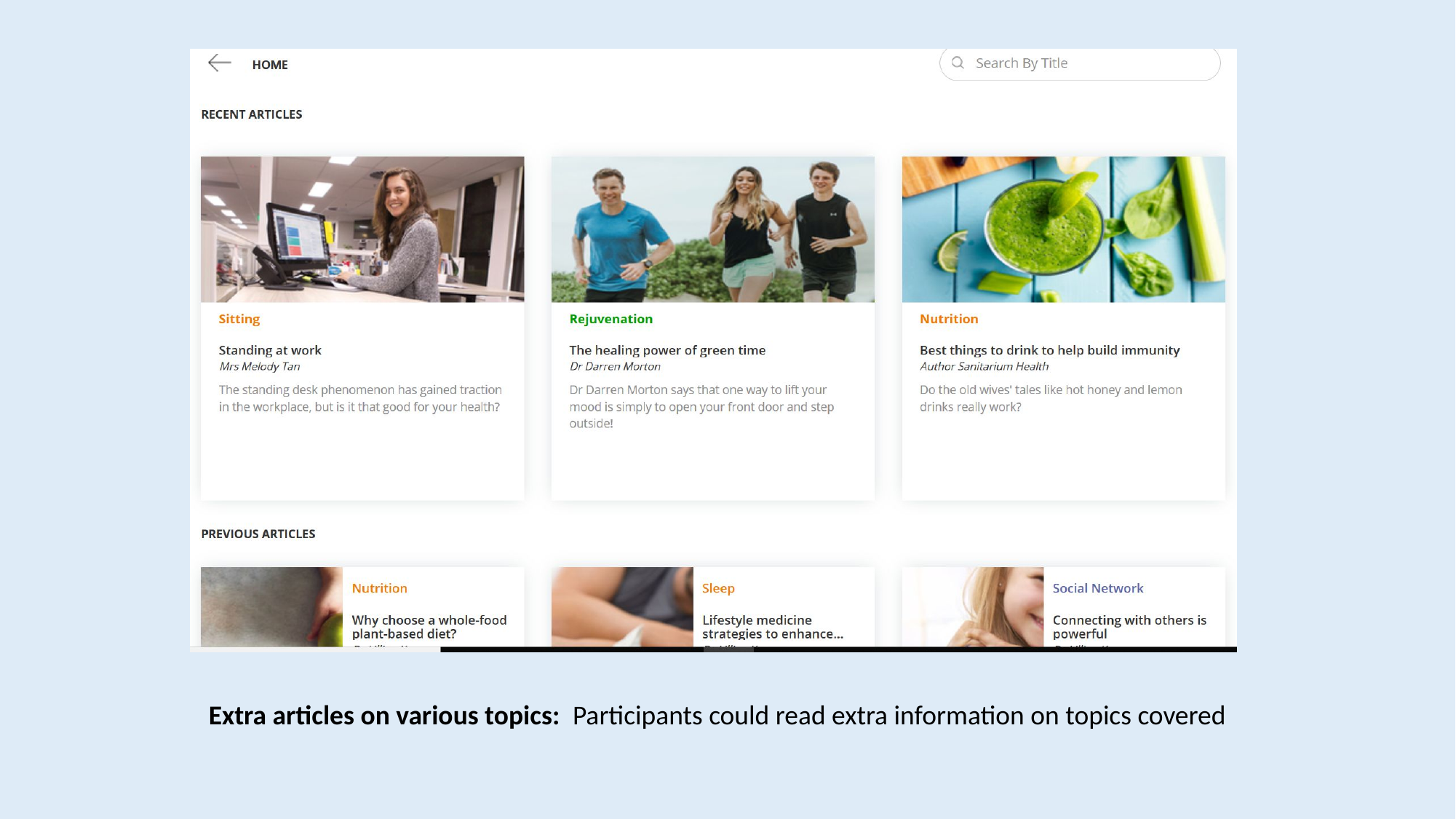

Extra articles on various topics: Participants could read extra information on topics covered

## Slide 10
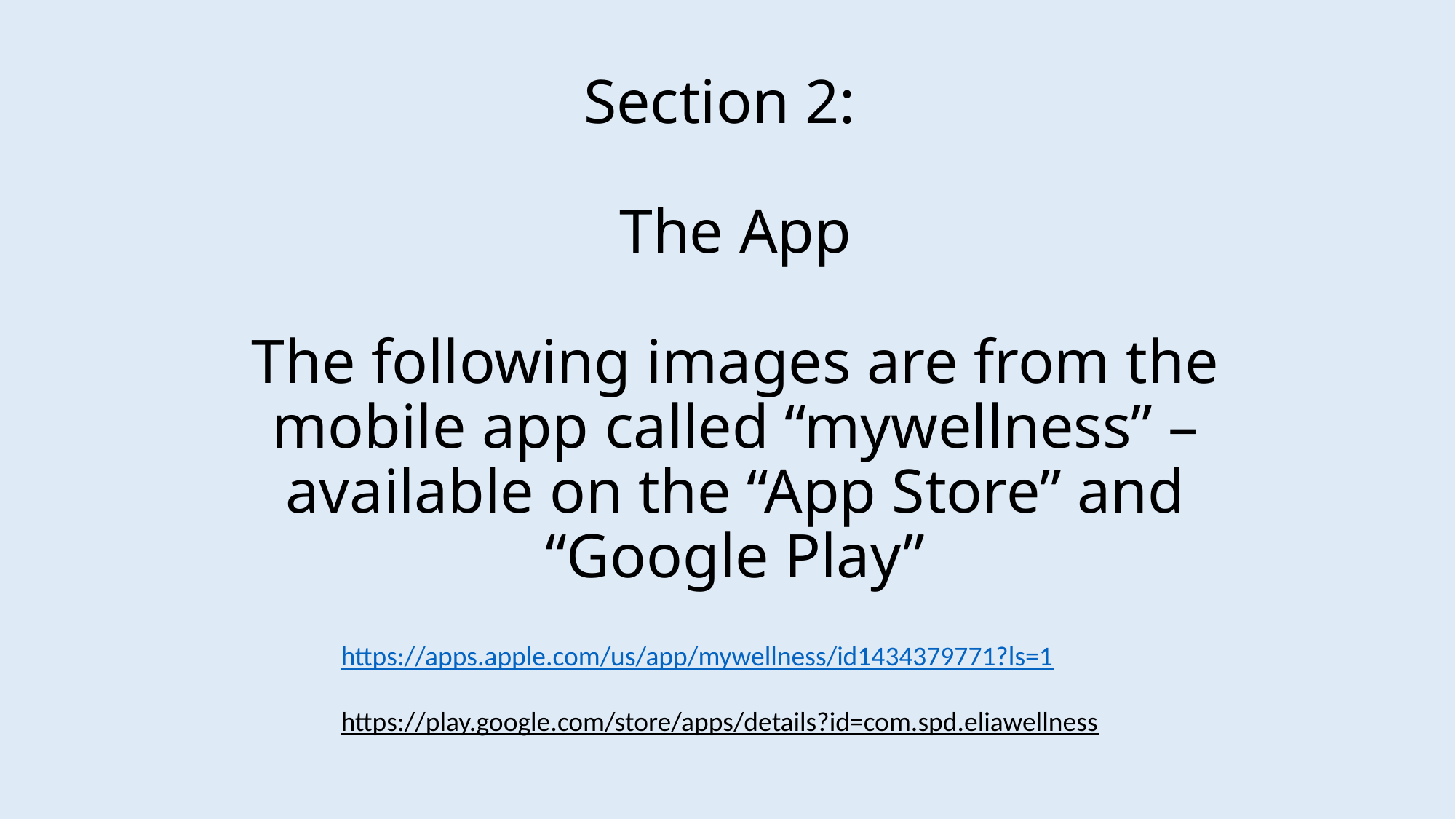

Section 2:
The App
The following images are from the mobile app called “mywellness” – available on the “App Store” and “Google Play”
https://apps.apple.com/us/app/mywellness/id1434379771?ls=1
https://play.google.com/store/apps/details?id=com.spd.eliawellness

## Slide 11
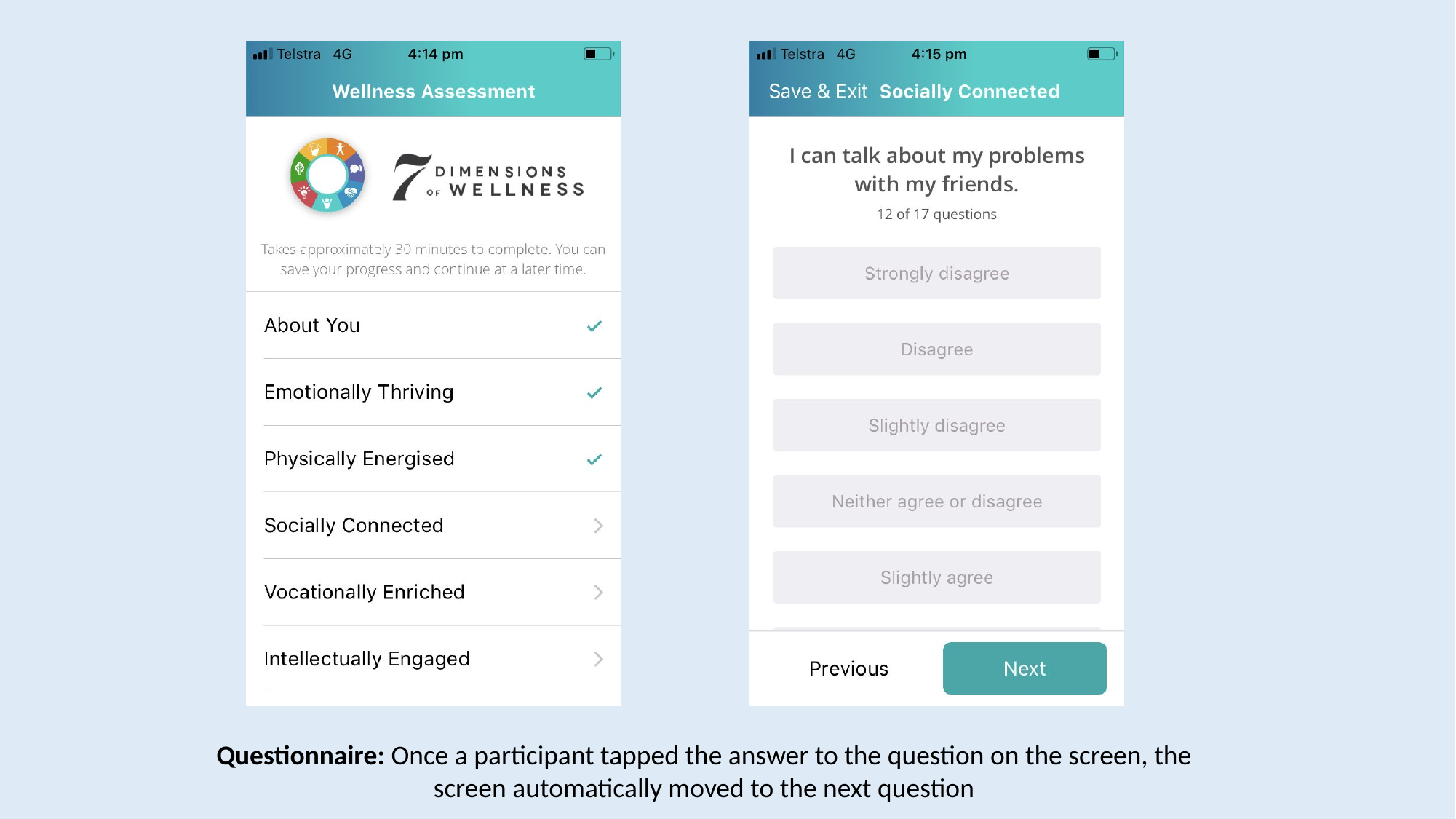

Questionnaire: Once a participant tapped the answer to the question on the screen, the screen automatically moved to the next question

## Slide 12
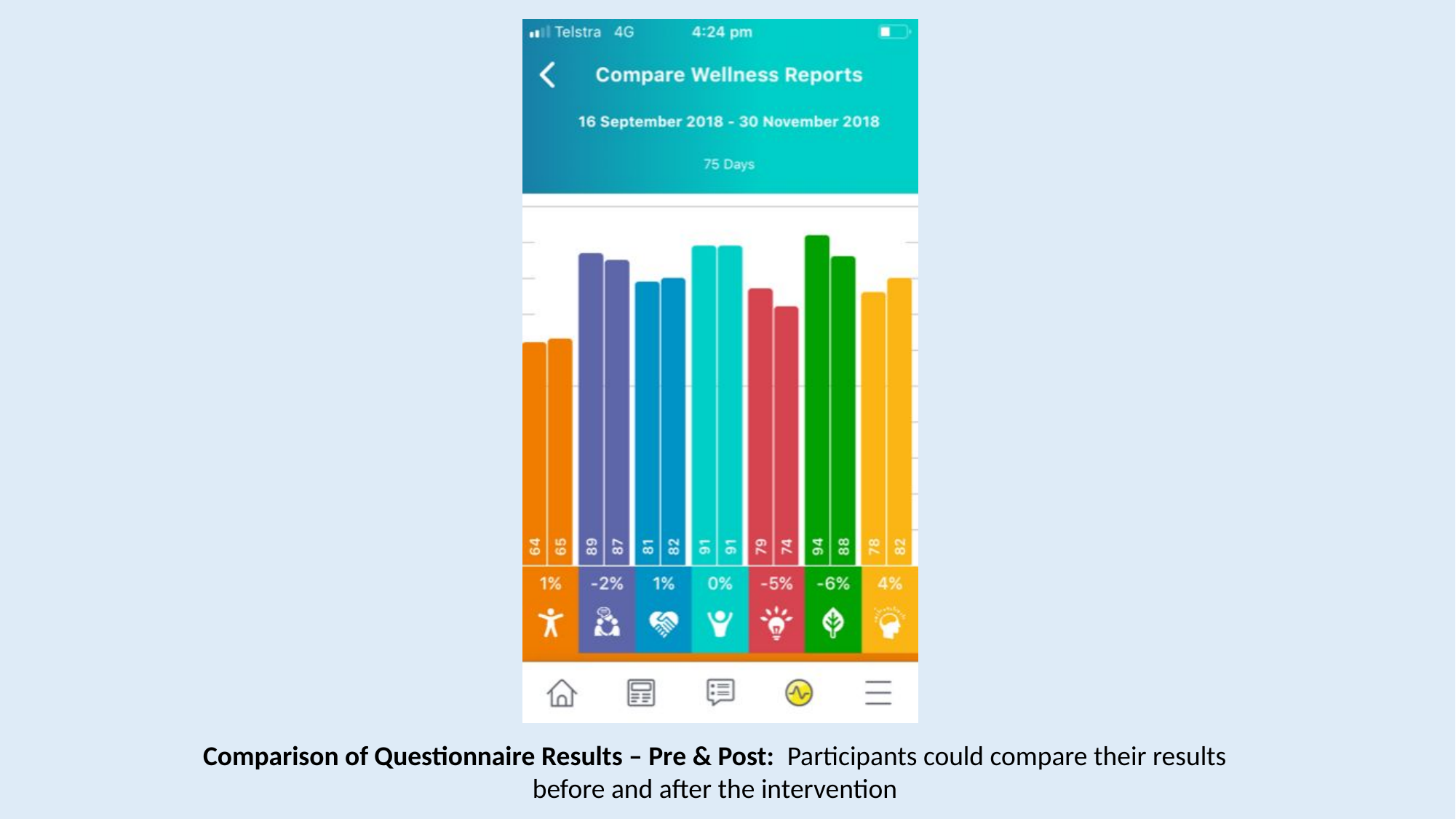

Comparison of Questionnaire Results – Pre & Post: Participants could compare their results before and after the intervention

## Slide 13
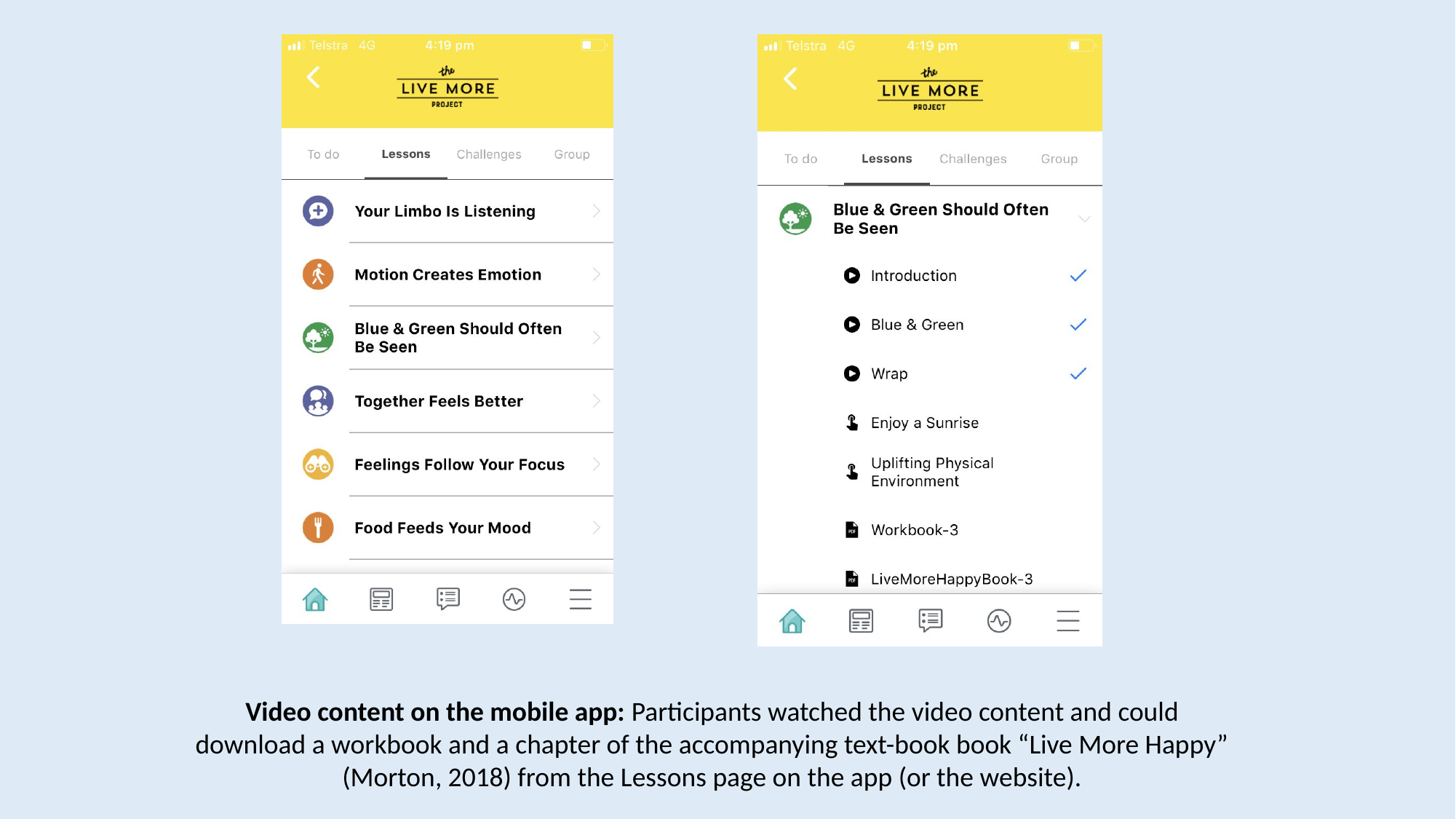

Video content on the mobile app: Participants watched the video content and could download a workbook and a chapter of the accompanying text-book book “Live More Happy” (Morton, 2018) from the Lessons page on the app (or the website).

## Slide 14
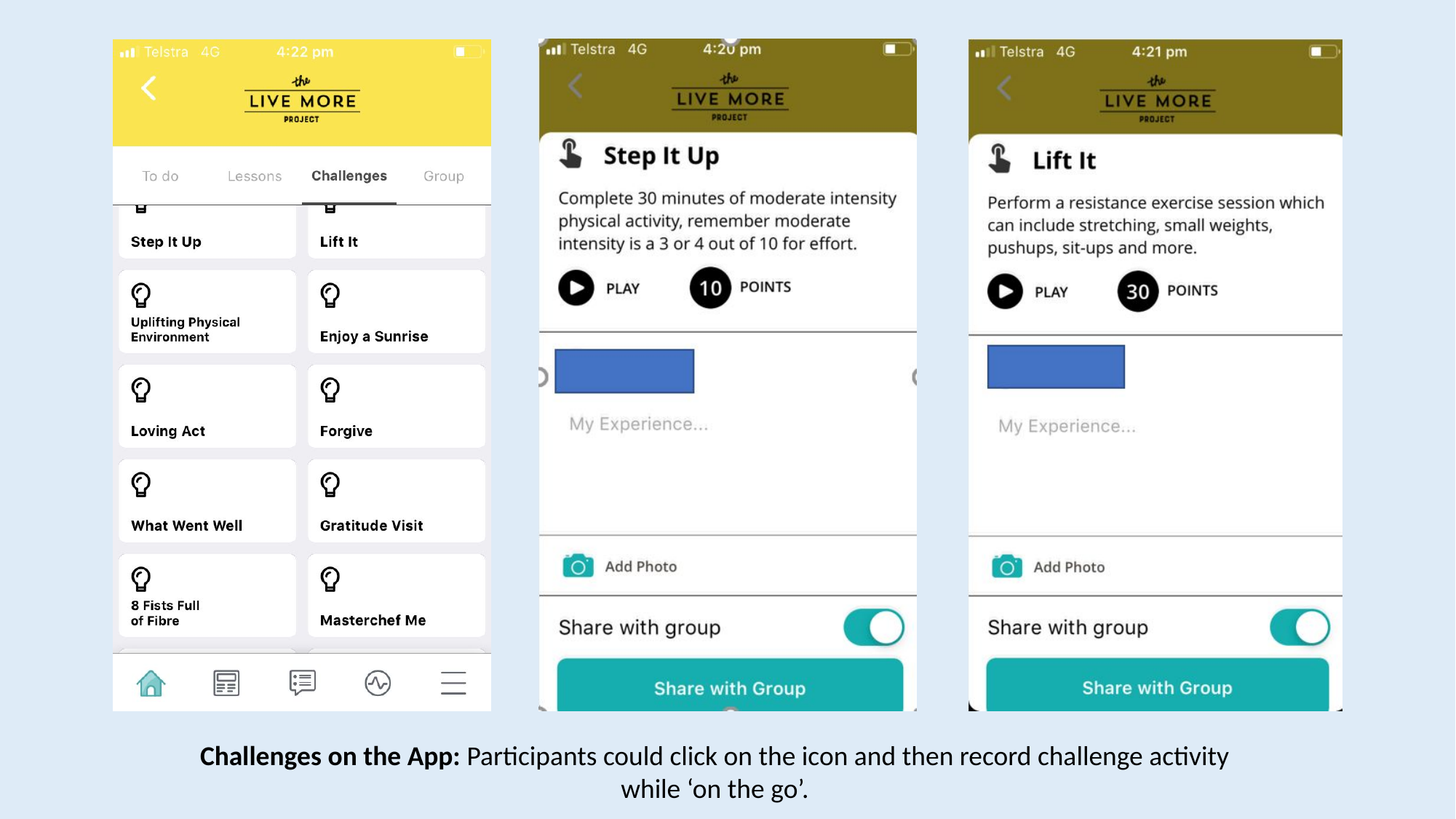

Challenges on the App: Participants could click on the icon and then record challenge activity while ‘on the go’.

## Slide 15
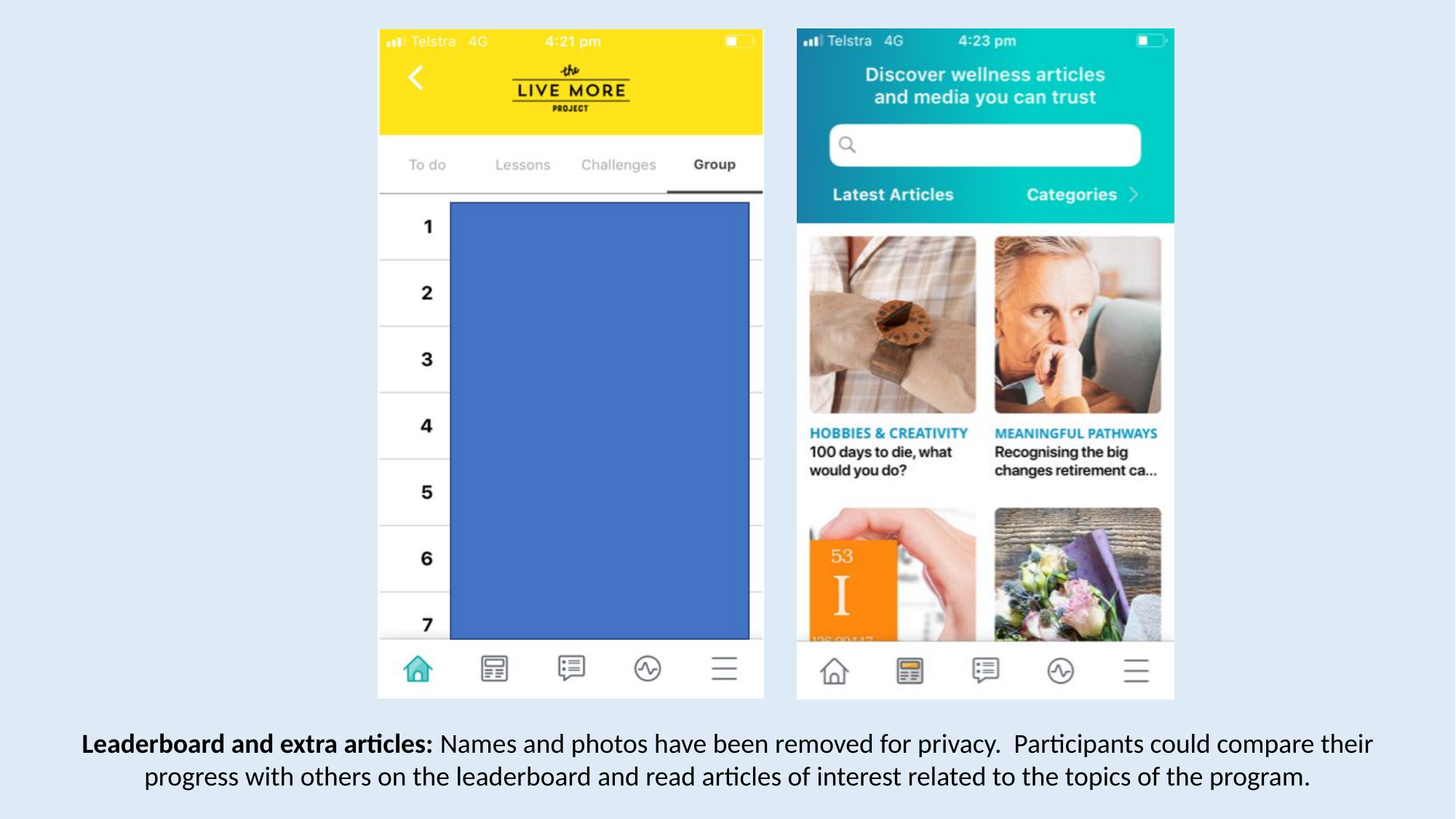

Leaderboard and extra articles: Names and photos have been removed for privacy. Participants could compare their progress with others on the leaderboard and read articles of interest related to the topics of the program.

## Slide 16
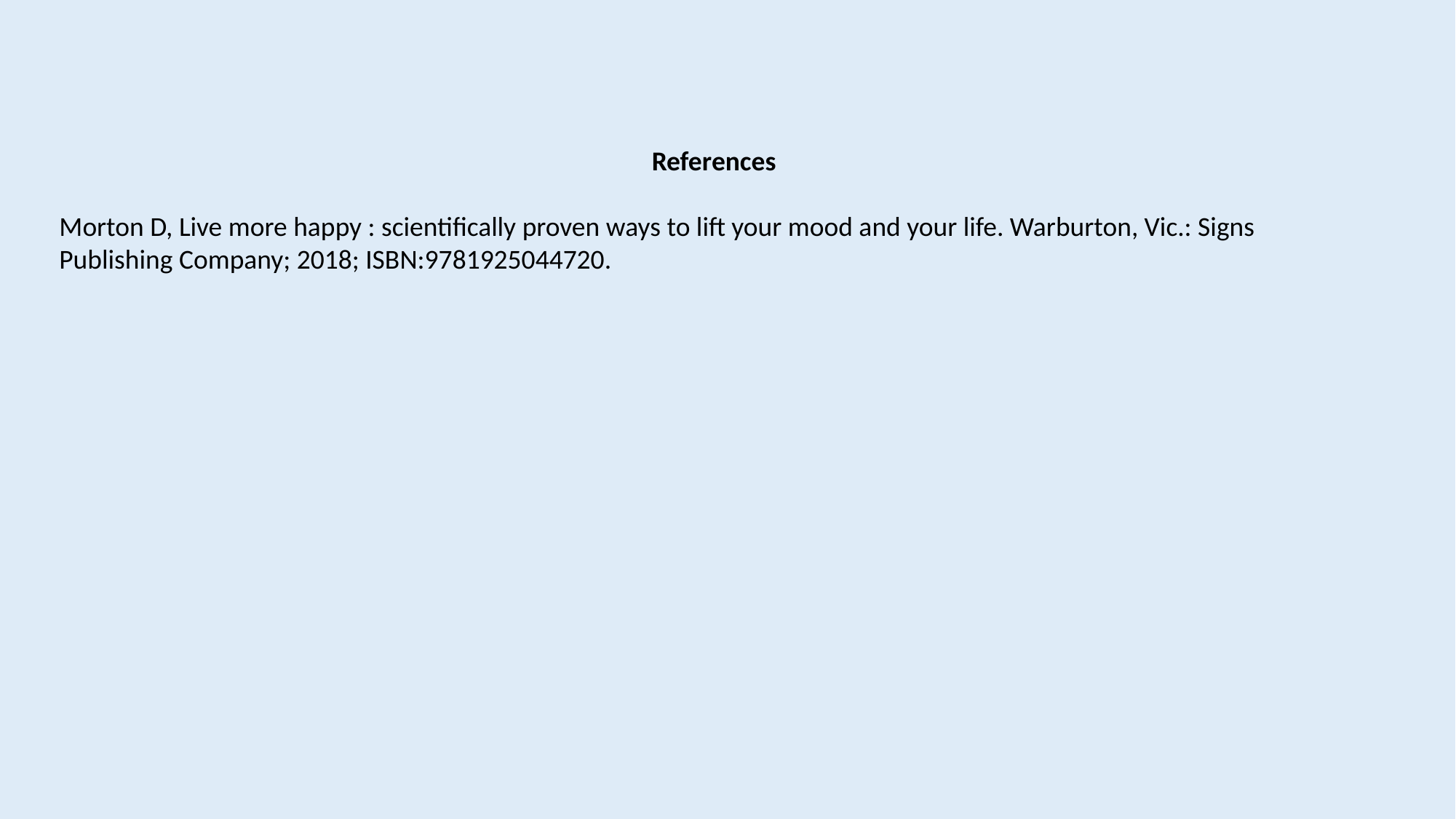

References
Morton D, Live more happy : scientifically proven ways to lift your mood and your life. Warburton, Vic.: Signs Publishing Company; 2018; ISBN:9781925044720.
