# Supplementary material for: A Web- and Mobile App–Based Mental Health Promotion Intervention Comparing Email, Short Message Service, and Videoconferencing Support for a Healthy Cohort: Randomized Comparative Study
Source: J Med Internet Res. 2020 Jan 6;22(1):e15592. doi: 10.2196/15592 (PMC6971514; doi:10.2196/15592)
Supplement: Multimedia Appendix 2 [file jmir_v22i1e15592_app2.docx]

Live More Project Research - Timetable

| Log on to the website address below to access videos & record your challenge activity [www.eliawellness.com/login](http://www.eliawellness.com/login) | | |
| --- | --- | --- |
| **Session Number** | **Week commencing** | **To Do** |
| **Orientation** | September 9 - 15 | Read “Invitation Email” (emailed 10/9/2018)  Register to gain access to the website  Log in & complete Questionnaire 1 |
| **Session 1** | September 16 - 22 | Watch video  Complete Daily & Weekly Challenges  Record challenges online |
| **Session 2** | September 23 - 29 | Watch video  Complete Daily & Weekly Challenges  Record challenges online |
| **Session 3** | September 30 – October 6 | Watch video  Complete Daily & Weekly Challenges  Record challenges online |
| **Session 4** | October 7 - 13 | Watch video  Complete Daily & Weekly Challenges  Record challenges online |
| **Session 5** | October 14 - 20 | Watch video  Complete Daily & Weekly Challenges  Record challenges online |
| **Session 6** | October 21 - 27 | Watch video  Complete Daily & Weekly Challenges  Record challenges online |
| **Session 7** | October 28 – November3 | Watch video  Complete Daily & Weekly Challenges  Record challenges online |
| **Session 8** | November 4 - 10 | Watch video  Complete Daily & Weekly Challenges  Record challenges online |
| **Session 9** | November 11 - 17 | Watch video  Complete Daily & Weekly Challenges  Record challenges online |
| **Session 10** | November 18 - 24 | Watch video  Complete Daily & Weekly Challenges  Record challenges online |
| **Questionnaire 2** | November 25 – December 9 | Complete Questionnaire 2 |
| **Questionnaire 3** | March 2019 | Complete Questionnaire 3 |
