# Supplementary material for: A Web- and Mobile App–Based Mental Health Promotion Intervention Comparing Email, Short Message Service, and Videoconferencing Support for a Healthy Cohort: Randomized Comparative Study
Source: J Med Internet Res. 2020 Jan 6;22(1):e15592. doi: 10.2196/15592 (PMC6971514; doi:10.2196/15592)
Supplement: Multimedia Appendix 3 [file jmir_v22i1e15592_app3.pdf]

You are invited to the Live More Project 'live' online meeting room...

The tables below include timetables for two periods: (1) No Daylight Saving & (2) During Daylight Saving

the LIVE MORE PROJECT

To find the online meeting times in your area:

1. *Locate your region*
2. *Read down that column to see the online group discussion times available for Sunday, Monday or Tuesday in your area*
3. *Choose a day & time that suits your schedule*
4. *Click on the link on your email or SMS to join the meeting at your chosen time (the 'room' will be open 10 minutes before meeting time)*

(1) Schedule of Zoom Online Group Discussions: September 16 - October 6 (NO DAYLIGHT SAVING)

	WA	NT	SA	QLD	TAS, VIC, ACT, NSW	NZ	*NZ (Sept 30 - Oct 6)
**Sunday**	3:00 PM	4.30 PM	4.30 PM	5:00 PM	5:00 PM	7:00 PM	8:00 PM
	4:00 PM	5.30 PM	5.30 PM	6:00 PM	6:00 PM	8:00 PM	9:00 PM
	6:00 PM	7.30 PM	7.30 PM	8:00 PM	8:00 PM	10:00 PM	11:00 PM
	7:00 PM	8.30 PM	8.30 PM	9:00 PM	9:00 PM	11:00 PM	MIDNIGHT
**Monday**	8:00 AM	9.30 AM	9.30 AM	10:00 AM	10:00 AM	NOON	1:00 PM
	3:00 PM	4.30 PM	4.30 PM	5:00 PM	5:00 PM	7:00 PM	8:00 PM
	5:00 PM	6.30 PM	6.30 PM	7:00 PM	7:00 PM	9:00 PM	10:00 PM
**Tuesday**	5:00 PM	6.30 PM	6.30 PM	7:00 PM	7:00 PM	9:00 PM	10:00 PM
	6:00 PM	7.30 PM	7.30 PM	8:00 PM	8:00 PM	10:00 PM	11:00 PM

* NZ Residents (note last column for 1 week period only : September 30 - October 6)

(2) Schedule of Live Zoom Meetings - From October 7 (DAYLIGHT SAVING TIME)

	WA	NT	SA	QLD	TAS, VIC, ACT, NSW	NZ
Sunday	3:00 PM	4.30 PM	5.30 PM	5:00 PM	6:00 PM	8:00 PM
	4:00 PM	5.30 PM	6.30 PM	6:00 PM	7:00 PM	9:00 PM
	6:00 PM	7.30 PM	8.30 PM	8:00 PM	9:00 PM	11:00 PM
	7:00 PM	8.30 PM	9.30 PM	9:00 PM	10:00 PM	MIDNIGHT
Monday	8:00 AM	9.30 AM	10.30 AM	10:00 AM	11:00 AM	1:00 PM
	3:00 PM	4.30 PM	5.30 PM	5:00 PM	6:00 PM	8:00 PM
	5:00 PM	6.30 PM	7.30 PM	7:00 PM	8:00 PM	10:00 PM
Tuesday	5:00 PM	6.30 PM	7.30 PM	7:00 PM	8:00 PM	10:00 PM
	6:00 PM	7.30 PM	8.30 PM	8:00 PM	9:00 PM	11:00 PM

Attend 1 online meeting per week

Pick a time in your region that suits you!

Meeting Link for Every Meeting: https://zoom.us/j/485170861
